# Functional social support and physical activity in community-dwelling older adults: a systematic review and meta-analysis

**DOI:** 10.1186/s12889-026-28972-z

**Published:** 2026-09-01

**Authors:** Paula Steinhoff, Lea Ellwardt, Amelie Reiner

**Affiliations:** 1 Department of Sociology and Social Psychology, University of Cologne, Cologne, Germany; 2 Institute of General Practice, Heinrich-Heine-University Düsseldorf, Düsseldorf, Germany

**Keywords:** Physical activity, Social support, Older adults, Systematic review, Meta-analysis

## Abstract

**Background:**

Physical activity (PA) is a key determinant of healthy ageing, yet a significant proportion of older adults remain insufficiently active. Functional social support (SOSU) – including emotional, instrumental, informational, companionship, and validation support – may facilitate PA engagement, but the strength and consistency of this association remain unclear.

**Methods:**

This study conducted a systematic review and meta-analysis to examine the relationship between functional SOSU and PA in community-dwelling older adults. The electronic databases APA PsycINFO, ProQuest, PSYINDEX, PubMed, Scopus, SocINDEX, and Web of Science were searched from date of database inception until 31 March 2026. A total of 54 peer-reviewed quantitative studies, representing a total of 252,206 participants, were included in the systematic review. 91% (*n* = 49) were cross-sectional and five were longitudinal studies. Thirty-three studies (55 models) contributed to the meta-analysis. Studies were assessed using the Newcastle–Ottawa Scale, and PRISMA guidelines were followed throughout. Forty-six studies were rated as good quality, seven as fair, and one as poor. Meta-analyses focused on general SOSU and social support specifically for PA (SSPA), using subjective PA outcomes.

**Results:**

Findings from the systematic review suggest a mostly positive association between functional SOSU and PA, particularly for companionship and emotional support. Meta-analysis revealed a moderate significant association between general SOSU and PA (β = 0.35, 95% CI: 0.16–0.53) and a small but significant association between SSPA and PA (β = 0.12, 95% CI: 0.06–0.17). Associations were strongest for light and leisure-time PA, and more consistent in women. Longitudinal studies, though limited, suggested a positive association between SOSU and PA that appeared to remain relatively stable over time.

**Conclusion:**

Functional SOSU appears to support PA in older adults, with general SOSU showing a stronger effect than SSPA. Future research should employ consistent, age-appropriate measurement tools. Additional longitudinal research is required to enable causal conclusions. Interventions integrating social support elements may improve PA uptake and promote healthier ageing.

**Supplementary Information:**

The online version contains supplementary material available at https://doi.org/10.1186/s12889-026-28972-z.

## Introduction

Both the number and total share of older adults are increasing worldwide. In 2022, the share of the world’s adult population aged 65 and over was approximately 10%; this share is projected to reach about 12% in 2030 and about 16% in 2050 [1]. Given these demographic trends, promoting healthy ageing is crucial, so as to enable well-being in older age.

A key factor in healthy ageing is physical activity (PA), defined as ‘any bodily movement produced by skeletal muscles that requires energy expenditure’ [2]. PA reduces not only all-cause mortality but also the risk of non-communicable diseases such as coronary heart disease, high blood pressure, stroke, type 2 diabetes, some types of cancers and depression. There is also strong evidence that regular PA improves functional health, cognitive function, mental health and overall quality of life [3, 4]. Nonetheless, 1.4 billion adults, 27.5% of the world’s adult population, do not meet the PA level recommended by the World Health Organization [2]. Physical inactivity is a global burden and is considered a pandemic. It is associated with high economic costs, as well as health and human costs [2, 5]. Among older adults, PA is particularly important because, in addition to its general health benefits, it reduces this population’s risk of falls, frailty and osteoporosis; its positive effect on physical function and cognitive health is also especially important during this life stage [6]. Longitudinal research highlights that even when PA is adopted later in life, it can still yield considerable significant health benefits for older adults [7]. The World Health Organization recommends that all older adults engage in PA and states that for substantive health benefits, this should amount to ‘at least 150–300 minutes of moderate-intensity aerobic physical activity; or at least 75–150 minutes of vigorous-intensity aerobic physical activity; or an equivalent combination of moderate- and vigorous intensity activity throughout the week’ [6].

Research has shown that receiving social support (SOSU) is associated with higher levels of PA in older adults [8, 9]. SOSU is a multidimensional concept and can be broken down into structural and functional elements. Structural SOSU refers to aspects such as the number of relationships providing SOSU and the subject’s geographical proximity to or frequency of interaction with these contacts, whereas functional SOSU tends to concern the quality of these relationships [10]. This paper focuses on functional SOSU, which consists of five dimensions, namely emotional SOSU, instrumental SOSU, informational SOSU, companionship SOSU and validation [11]. Emotional support can be defined as the availability of an individual who is willing to listen to, care for, accept, encourage and appreciate the person in need of such support. In contrast, instrumental support is more concrete in nature and encompasses the provision of practical assistance. Informational support refers to the provision of information about resources and knowledge valuable for coping with problems, as well as counselling and guidance. Companionship support concerns the presence of people with whom the individual can engage in social and leisure activities. The final dimension, often referred to as validation, feedback or social comparison, is based on the idea that social relationships can provide information about the conformity or appropriateness of behaviours [11, 12]. As a whole, the concept of functional SOSU suggests that personal relationships offer different types of SOSU and that the importance and effectiveness of each type can vary depending on the specific problem or challenge being faced. This concept is appropriate in older adults, as the different dimensions of functional SOSU act as different facilitators of and barriers to PA [9, 12].

### Aim of the current research

Although existing findings are mixed, research has generally indicated a positive association between functional SOSU and PA in older adults [8, 9]. However, important gaps remain in the literature. The only systematic review to have examined this topic is Lindsay Smith et al. [8], who reviewed 27 quantitative studies on the association between SOSU and PA in community-dwelling older adults aged 60 and over. Their review found a positive association between SSPA and PA levels, particularly when support came from family members, while the evidence for general SOSU and PA remained unclear. However, that review has two critical limitations that constrain the conclusions that can be drawn today. First, its literature search was completed in August 2014, meaning that a decade of research – including the considerable volume of studies produced in the wake of the COVID-19 pandemic – has not been captured. Second, and importantly, Lindsay Smith et al. conducted a narrative synthesis only; without a meta-analysis that pools effect sizes the overall magnitude of the association between SOSU and PA in older adults remains unknown.

The most recent relevant work is a scoping review by Steinhoff and Reiner [9] which provides a comprehensive and up-to-date descriptive overview of the topic. This review incorporated qualitative and mixed-methods studies alongside quantitative evidence, with 22% of the included studies employing a qualitative design and 6% a mixed-methods design. It addressed a broad research question, examining the association between functional SOSU and PA in both directions and treating both SOSU and PA as either a predictor or an outcome. While this approach provides an extensive overview of the available evidence without excluding relevant studies based on methodology – which a systematic review focusing solely on quantitative evidence would not capture – a scoping review maps the scope of the evidence, rather than evaluating its quality or quantifying effect sizes. Consequently, it neither assesses risk of bias nor produces pooled estimates. As a result, it is not possible to draw firm conclusions regarding the consistency and magnitude of the association.

While the scoping review provided an initial overview of the field, this study takes further steps: First, it aims to systematically review and summarise quantitative evidence on the association between functional SOSU and PA in community-dwelling older adults, restricting the direction of association to functional SOSU as the independent variable and PA as the dependent variable; second, this paper aims to quantify this association through meta-analyses, producing pooled effect sizes that have not previously been reported for this population. Additionally, applying statistical techniques enables an evaluation of potential publication bias, thereby strengthening the robustness of the conclusions drawn [13].

This study is guided by the following research questions:

- (1) How does functional SOSU affect PA in community-dwelling older adults?
- (2) What is the overall magnitude of the association between functional SOSU – specifically SSPA and general functional SOSU – and PA in older adults? How do these associations vary by PA type?

By addressing these questions, this study moves beyond descriptive mapping to offer a rigorous, quantitative synthesis that can inform evidence-based policy, intervention design and future research aimed at promoting PA in older adults.

## Methods

This systematic review and meta-analysis build on the scoping review, conducted by Steinhoff & Reiner [9]. The original search covered the period from database inception through August 2023 and resulted in the inclusion of 116 articles. These 116 articles formed the basis of the present review. In addition, an updated literature search covering the period from August 2023 to 31 March 2026 was conducted using the same search strategy as applied in the scoping review. However, we adapted the selection process from the scoping review for this systematic review and meta-analysis. For the present study, we followed the PRISMA guidelines for the procedures to be employed in systematic reviews and meta-analyses [14]. The systematic review and meta-analysis were preregistered; the protocol can be accessed at https://osf.io/uc5xe.

### Eligibility criteria

We included cross-sectional and longitudinal peer-reviewed empirical studies in English that examined the association between functional SOSU and PA in older populations. In line with the WHO definition, older adults were defined as individuals aged 60 years and older [15].

We did not set any chronological or geographical restrictions. We only included studies in which SOSU was the independent variable and PA was the dependent variable. The association between functional SOSU and PA was required to be reported through multivariate analysis, whilst controlling for confounders established during quality assessment.

A minimum participant age criterion of 40 years was opted for to allow the inclusion of relevant ageing studies, such as the German Ageing Survey (DEAS). To retain a focus on older adults specifically, studies were required to report a mean participant age of at least 60 years, consistent with the WHO definition of older adults.

We excluded experimental studies, qualitative studies, mixed methods studies reporting only qualitative findings, editorials, commentaries, study protocols, conference proceedings, grey literature and literature reviews. We also excluded studies that focused only on structural aspects of SOSU and did not measure functional aspects. Furthermore, we excluded studies that did not specifically measure moderate-to-vigorous PA (MVPA), leisure-time PA (LTPA), light PA (LPA) or total PA (TPA) or that only reported PA levels using a health-related fitness measure. Lastly, studies focusing exclusively on specific clinical patient populations or institutionalised or hospitalised persons were excluded.

Studies included in the systematic review were not automatically eligible for the inclusion of the meta-analysis: In the systematic review section of this article, we identify and summarise studies that address all dimensions of functional SOSU as well as studies employing at least one dimension of functional SOSU that cannot be clearly assigned to one of these five dimensions, or that cover functional SOSU without analysing its dimensions separately (general SOSU). Additionally, we include studies which look specifically at functional SOSU for PA activity (SSPA). For the meta-analysis, however, only studies assessing general SOSU or SSPA were included. This was due to the limited number of studies addressing the other dimensions of functional SOSU, which would have prevented meaningful statistical comparison. Furthermore, considerable heterogeneity was observed across these studies with respect to the measurement of both the independent and dependent variables. By contrast, the instruments used to assess general SOSU or SSPA were more homogeneous and thus more suitable for statistical comparison. In addition, the meta-analysis was restricted to studies assessing subjective PA, as too few studies used objective PA measures to permit meaningful statistical analyses. Furthermore, for inclusion into the meta-analysis studies were required to provide sufficient information to enable the calculation of effect sizes and measurement error. Consequently, studies were excluded if they did not report the effect size or error measure directly or provide enough information to derive them. For example, when a standardised regression coefficient (β) was reported without the standardised standard error (SE), the latter could be derived from the t-value – if reported. Where information was missing, the corresponding authors were contacted to request it; however, not all of them responded. Lastly, studies required that reported effect sizes deem reliable and comparable across studies to permit meaningful pooling. Studies were excluded from the meta-analysis where standardised regression coefficients exceeded ± 1, as they reflect statistical artefacts of predictor intercorrelation rather than true effect magnitudes. Such studies were retained in the narrative synthesis, but should be interpreted cautiously.

### Information sources and search strategy

In this systematic review, we used data from a previously completed scoping review [9]. The original literature search was carried out on material published up to 8 August 2023. To ensure that we included all available evidence for this article, we conducted a follow-up literature search covering the period from August 2023 to 31 March 2026. Searches were conducted in the databases PubMed, Web of Science, Scopus, APA PsycInfo, ProQuest, SocINDEX and PSYINDEX, as well as in the Cochrane Library from database inception to March 31, 2026. Additionally, reference lists from other reviews were examined to uncover any further relevant studies. Initial search terms (Supplementary Material 1) were created to address the three key aspects of the research focus: ‘older adults’, ‘social support’ and ‘physical activity’. For the search strategy, we first reviewed terms used in previously published reviews [8, 16]; subsequently, a multidisciplinary team of researchers PS (health scientist), AR and KH (demographers and sociologists specialising in ageing) and LE (sociologist with expertise in social network research, health and ageing) collaborated to refine and expand the search terms.

### Selection process

The results of the database searches were imported into Rayyan [17] for management, where duplicate entries were removed. Two researchers (PS and AR) independently screened the resulting titles and abstracts while maintaining blinding. They then separately assessed the full text of apparently relevant studies against the eligibility criteria. Any disagreements were resolved through in-depth discussion to reach a consensus. To ensure reliability, PS and AR conducted two pilot screenings with a random selection of 100 studies for each pilot.

### Data collection process and data extraction

PS and LE independently extracted the data into a table whose structure was informed by other reviews [8, 16]. For each included article, they extracted bibliographic details and, for every model examining the association of interest, recorded information on (a) the sample (year of data collection, percentage of male participants, mean age, sample size, dataset type and any subsample used), (b) the independent and dependent variables (including type and measurement instrument), (c) the analytical approach, (d) the results (e.g. coefficients, error measures, means and standard deviations) and (e) the number of control variables. The primary outcome was PA levels in community-dwelling older adults. Any disagreements during data extraction were resolved through discussion with a third researcher (AR).

### Data items

In this systematic review, we specifically included moderate-to-vigorous physical activity (MVPA), leisure-time physical activity (LTPA), total physical activity (TPA) and light physical activity (LPA) to encompass the full range of PA behaviours in older adults. Moreover, these four categories are incorporated into most standard PA measurement scales. In terms of functional SOSU, we included studies which measured at least one of the five dimensions of functional SOSU [11].

### Risk of bias and quality assessment

The Newcastle–Ottawa Scale (NOS) for cross-sectional and cohort studies [18] was used to assess the quality of the studies considered. Two researchers (MW and PS) conducted the assessment independently. The Newcastle–Ottawa Scale assesses the quality of a study by evaluating its design, the way in which participants are selected, how comparable it is and how it assesses exposure and outcomes. It assigns stars for each category, with more stars indicating higher quality. In line with Agency for Health Research and Quality standards, we categorised the studies as being of low, moderate or good quality. A risk of bias assessment was conducted to inform the interpretation of findings but was not used to exclude studies or assign weights. This approach is in line with previous systematic reviews [19–21].

### Synthesis methods and analytical strategy

For the systematic review, the results are reported narratively according to the type of functional SOSU considered. The findings are categorised into the following functional SOSU dimensions: emotional SOSU, instrumental SOSU, informational SOSU, companionship SOSU, validation and SOSU for PA (SSPA). Studies that used items covering functional SOSU but did not analyse the dimensions separately are included under ‘general SOSU’.

Studies using instruments that combine functional and structural items into a single composite score – such as the total score of the Duke Social Support Index (DSSI) – were included under the general SOSU category, given that the DSSI is a validated and reliable measure of SOSU in older adults and its Satisfaction/Subjective Support subscale captures functional SOSU. However, studies that used validated instruments for general SOSU, but reported results based exclusively on subscales that only captured structural aspects of SOSU (such as the DSSI Social Interaction subscale) were excluded.

For each category of functional SOSU, a concise data extraction table is provided, summarising key study details including the first author, year, continent, study design, mean age, sample size, SOSU and PA measures, significance of results and quality rating. In these tables, studies are labelled with ‘+’ or ‘−’ to represent statistically significant positive or negative associations, respectively; ‘0’ denotes no significant association, and ‘*’ is used when both significant positive and significant negative associations were reported. We subsequently analysed the findings across all functional SOSU types to identify differences between cross-sectional and longitudinal studies, as well as variations based on PA measurement, PA domain, gender and SOSU source.

For the meta-analysis, we present four forest plots, one for general SOSU with a continuous PA outcome (11 models), one for general SOSU with a binary PA outcome (7 models), one for SSPA with a continuous PA outcome (28 models) and one for SSPA with a binary PA outcome (9 models). We further conduct random-effects meta-regression analysis to explore whether the pooled effect size varied according to a number of covariates and discuss the risk of publication bias. These covariates concerned the percentage of male subjects in the sample (if no information was provided, we imputed results based on a 50% male sample), mean age (if only age categories were provided, we imputed mean age using subsample sizes), region (i.e. continent), type of PA (LTPA = ref., MVPA, LPA, other PA) and source of support (general = ref., family, friends). Due to the small number of studies, we refrained from including all covariates in a single model to avoid overfitting. The meta-regression analysis included 35 models and used dummy variables to account for the nestedness of models within studies; additionally, we adjusted for type of support (general SOSU versus SSPA).

## Results

**Fig. 1:**
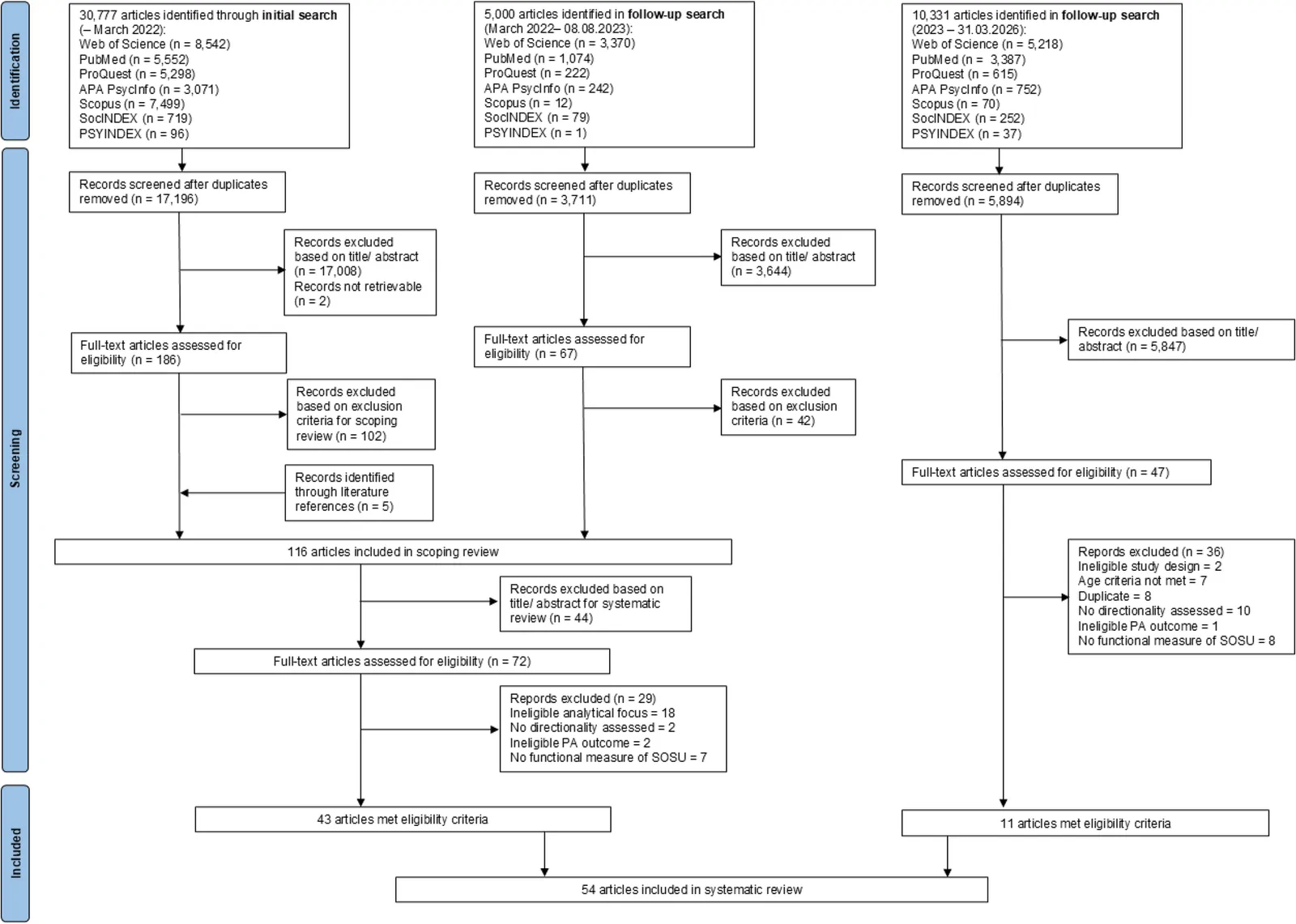
PRISMA-P flowchart

This systematic review draws directly on the 116 articles identified in a scoping review previously conducted by Steinhoff and Reiner [9], as well as on a subsequent literature search conducted after the scoping review, covering the period from August 2023 to 31 March 2026. For the current review, we applied additional exclusion criteria to those 116 articles, resulting in the removal of 38. The remaining 78 full-text articles were reassessed, with 43 meeting the eligibility criteria for inclusion in this systematic review. In the follow-up literature search, we identified a total of 10,331 articles. After removing duplicates, we screened 5,894 articles for eligibility based on their titles and abstracts. We included 47 articles in the full-text screening stage of which eleven fulfilled the eligibility criteria and were retained for analysis. Consequently, this systematic review includes a total of 54 articles. The complete process is visually represented in the PRISMA-P flowchart in Fig. 1.

### General study characteristics

An overview of the characteristics of the included studies is provided in Table 1. Of the 54 studies, 90.74% (*n* = 49) were cross-sectional and five had a longitudinal design. Studies were published between 1993 and 2025, with more than two-thirds (*n* = 37, 68.5%) being published after 2015. This makes the present study an important update to the systematic review by Lindsay-Smith et al. [8].

**Table 1:** Summary characteristics of included studies (*N* = 54)

Characteristics	*N*	%
Study design		
Cross-sectional	49	90.7
Longitudinal	5	9.3
Region		
North America	22	40.7
Asia	18	33.3
Europe	8	14.8
South America	4	7.4
Australia	1	1.9
Europe & Asia	1	1.9
Publication period		
Before 2000	3	5.6
2000–2009	6	11.1
2010–2019	22	40.7
2020–2025	23	42.6
Mean age of participants		
Young-old (60–69 years)	25	46.3
Old-old (70–79 years)	27	50.0
Not reported	2	3.7
Sample size		
< 200	10	18.5
200–999	20	37.0
1,000–9,999	16	29.6
≥ 10,000	8	14.8
PA measurement		
Subjective only	50	92.6
Objective only (accelerometery)	3	5.6
Subjective and objective (combined)	1	1.9
Subjective PA measurement		
Standardised scale	31	57.4
Study-specific items	23	42.6
SOSU measurement		
Standardised scale	25	46.3
Study-specific items	29	53.7
Methodological quality (NOS)		
Good	46	85.2
Fair	7	13.0
Poor	1	1.9

*NOS* Newcastle–Ottawa Scale, *PA* Physical activity, *SOSU* Social support. Mean ages of study samples ranged from 60.0 to 79.95 years (median: 70.1 years); two studies did not report mean age. Total participants across all included studies: *N* = 252,206. Sample sizes ranged from 36 to 78,002

Most of the included studies (*n* = 46) were rated as good quality. Seven studies were classified as fair quality and one as poor quality. The findings appeared consistent across studies of varying quality levels.

The greatest number of studies were carried out in North America (*n* = 22, 40,7%), followed by Asia (*n* = 18, 33%). Eight studies were performed in Europe (14,8%) and one in both Europe and Asia [22]. Four studies were conducted in South America, and only one was carried out in Oceania. We did not identify any studies from Africa. Although the results appeared stable across geographic regions, this imbalance must be considered, as the results may not be generalisable to all populations. The mean age of study participants varied from 60 to 79.95 (median: 70.1 years). Twenty-five studies fell into the 60–69 age category and 27 into the 70–79 age category. Two studies did not report a mean age. Five studies focused exclusively on women [23–27] and one exclusively on men [28]. Seven studies examined gender differences in the relationship between functional SOSU and PA [29–35]. Sample sizes ranged from 36 [36] to 78,002 [37] respondents.

PA can be measured either subjectively (using self-report) or objectively (using device-based data or reports from external persons). Approaches to measuring and analysing PA differed considerably among the included studies: 47 (87%) examined subjective PA only, three combined subjective and objective PA [38–40], two investigated both objective and subjective PA [41, 42] and two focused on objective PA only [35, 43]. Most studies reported continuous PA outcomes (67%, *n* = 36), while 24% (*n* = 13) used binary outcomes and 9% (*n* = 5) collected categorical data. There was considerable variation in how SOSU was measured, ranging from single-item questions to the use of validated scales such as the Duke Social Support Index or the Social Support for Exercise Scale (SSES). In terms of dimensions, 13 studies explored general SOSU, 25 examined SSPA, 10 investigated emotional SOSU, 2 studied informational SOSU, 7 focused on instrumental SOSU and 6 explored companionship SOSU. None of the included studies examined the relationship between validation and PA.

### Differences in study design

Of the included studies, 90.74% (*n* = 49) were cross-sectional, which limits the resulting potential for conclusions about causality. Although most of these studies reported significant positive associations between functional SOSU and PA, several found no significant relationship, and a few even reported negative associations. Two studies employed an Ecological Momentary Assessment (EMA) approach with a daily diary design. In these studies, participants completed evening questionnaires for up to ten consecutive days. Despite having a repeated-measure design and covering a period of ten days, we have decided to treat these EMA studies as cross-sectional due to significant differences in temporal scope, research questions, and findings, which speak to within-person changes rather than long-term change. Of these studies, one found a significant positive association between SOSU and PA [40], while the other found no significant association [39]. The five longitudinal studies provided stronger evidence of associations between SOSU and PA over time: four identified significant positive associations [44–46], while one found no significant relationship [32].

Despite their limited number, the longitudinal studies largely suggested a relatively stable positive association between SOSU and PA over time. In one study, for example, SOSU from friends at baseline was significantly associated with physical exercise frequency six months later [46], and other studies reported significant positive associations after three [25], eight [25], nine [45] and twelve years [44]. However, the overall mixed findings highlight the need for additional longitudinal and experimental research to clarify causal relationships between SOSU and PA.

### General SOSU

In total, eleven cross-sectional studies and two longitudinal studies [25, 44] measured the association between PA and general SOSU (see Table 2). Of these studies, twelve measured subjective PA [25, 26, 30, 31, 44, 47–53], and one study measured objective PA [43]. Almost all studies showed a significant positive association between general SOSU and PA, with only three exceptions: one study observed no significant association [52] and two reported a significant negative association [26, 48], all of which concerned subjective PA. Interestingly, Gomes et al. discovered that receiving SOSU had a significant negative association with PA, while providing SOSU was significantly positively associated with PA [48]. This suggests that individuals who require support may be less capable of engaging in PA than those who can provide it.

**Table 2:** Overview of results: general social support and physical activity

Author, year	Continent	Study design ^a^	Mean age	*N* ^b^	SOSU measure	PA type ^c^	PA measure	Results ^d^	Quality
Blakoe et al., 2023 [31, 48]	Asia	CS	67.34	3,969	DSSI	S	GPAQ	+	Good
Chen et al., 2021 [44]	Europe	CS	72.63	595	9 items	O	Accelerometer	+	Good
Gomes et al., 2017 [49]	Europe	CS	67.8	19,298	2 items	S	2 items (PA frequency)	*	Good
Harvey & Alexander, 2012 [26]	North America	L	69	671	6 items	S	3 items	+	Good
Hwang & Yi, 2025 [50]	Asia	CS	60.24	974	MSPSSS	S	GLTEQ (3 items)	+	Good
Jin et al., 2022 [51]	Asia	CS	69	778	SSRS	S	IPAQ-SF	+	Good
Kang et al., 2018 [52]	Asia	CS	69.28	332	SSQ	S	GLTEQ	+	Good
Kaplan et al., 2001 [32]	North America	CS	72.25	12,611	4 items	S	1 item (LTPA)	+	Good
Lieber et al., 2024 [53]	North America	CS	70.1	178	ISEL-12	S	GPAQ	0	Fair
Marthammuthu et al., 2023 [27]	Asia	CS	70.83	1,221	DSSI	S	PASE	-	Good
Manz et al., 2018 [45]	Europe	L	60	1,143	1 item (from Oslo-3 Social Support Scale)	S	1 item (LTPA)	+	Good
Yi et al., 2016 [54]	Asia	CS	67.11	1,580	5 items	S	3 items	+	Good

Social support measures: *DSSI* Duke Social Support Index, *ISEL-12* Interpersonal Support Evaluation List, *MSPSS* Multidimensional Scale of Perceived Social Support, *SSRS* Social Support Rate Scale, *SSQ* Perceived Social Support Questionnaire

Physical activity measures: *GLTEQ* Godin’s Leisure Time Exercise Questionnaire, *GPAQ* Global Physical Activity Questionnaire, *IPAQ* International Physical Activity Questionnaire, *PASE* Physical Activity Scale for the Elderly

^a^*CS* Cross-sectional studies, *L* Longitudinal studies

^b^*N* Sample size

^c^*S* Subjective PA, *O* Objective PA

^d^ Results: 0 indicates no sig. relationship (*p* ≥ 0.05), + indicates sig. pos. relationship (*p* < 0.05), - indicates sig. neg. relationship, * indicates sig. mixed relationship

Evidence on gender differences in the relationship between general SOSU and PA was inconclusive. When the association was examined separately for each gender, significant positive associations were found among women but not among men [30, 31]. This suggests that SOSU may play a more important role in encouraging PA among women. Among studies focusing exclusively on female participants, one found a negative association between general SOSU and PA [26], while another identified a positive link between friend-specific general SOSU and PA but no significant effect from family support [25].

### SSPA

Over 50% (*n* = 25) of the included studies analysed the relationship between SSPA and PA (see Table 3). The results were, as a whole, inconclusive. However, the studies concerned also varied in terms of the number of participants (n_min_ = 36 versus n_max_ = 3,692), the PA outcomes (e.g. objective PA, subjective PA, TPA, park-based PA) and the mean age of participants (64.8–74.4). Of these 25 studies, 13 (50%) employed some version of the SSES [54].

**Table 3:** Overview of results: social support for physical activity and physical activity

Author, year	Continent	Study design ^a^	Mean age	*N* ^b^	SOSU measure	PA type ^c^	PA measure	Results ^d^	Quality
Abdi & O’Hern, 2025 [30]	Asia	CS	60	3,692	1 item	S	1 item	+	Good
Bakhtari et al., 2019 [58]	Asia	CS	69.17	340	20 items	S	PASE-SF	+	Good
Bopp et al., 2004 [24]	North America	CS	70.6	102	SSES	S	PASE	+	Good
Carlson et al., 2012 [42]	North America	CS	74.4	707/709	SSES (adapted)	S	CHAMPS	+	Good
Carlson et al., 2012 [42]	North America	CS	74.4	707/709	SSES (adapted)	O	Accelero-meter	+	Good
Corseuil Giehl et al., 2017 [77]	South America	CS	70.3	1,705	SSES	S	IPAQ (walking domain)	+	Good
Cousins, 1996 [25]	North America	CS	76.7	136	4 items	S	1 item?	+	Fair
de Sousa et al., 2021 [78]	South America	CS	69.9	208	SSPAS	S	IPAQ	0	Good
Gothe, 2018 [39]	North America	CS	64.8	110	SSES	All PA	Accelero-meter, PASE, GLTEQ merged	0	Good
Kim & Kosma, 2013 [65]	Asia	CS	68.56	290	SSES (adapted) Family	S	GLTEQ	+	Good
Lee & Fan, 2023 [61]	Asia	CS	71.41	183	5 items	S	PASE	0	Good
Lian et al., 1999 [34]	Asia	CS	NR	1052	1 item (family PA support)	S	Items on weekly MVPA frequency	+	Good
Newsom et al., 2018 [71]	North America	CS	72.55	217	4 items	S	CHAMPS	0	Good
Oka & Shibata, 2012 [36]	Asia	CS	74.5	137	5 items	O	Accelero-meter	0	Fair
Orsega-Smith et al., 2007 [62]	North America	CS	67.7	1,900	SSES	S	2 items (LTPA)	+	Good
Park et al., 2014 [56]	Asia	CS	71.62	187	SSES (family)	S	PASE	-	Good
Safavi et al., 2025 [63]	Asia	CS	65.89	550	SSES	S	IPAQ-SF	0	Good
Sirotiak et al., 2024 [66]	North America	CS	68.55	95	SSES	S	PASE	+	Fair
Steijvers et al., 2025 [64]	Europe	CS	68	1,975	1 item	S	IPAQ-SF	+	Good
Thornton et al., 2017 [43]	North America	CS	74.4	726	4 items	O	Accelero-meter	+	Good
Thornton et al., 2017 [43]	North America	CS	74.4	726	4 items	S	CHAMPS	+	Good
van Luchene et al., 2021 [37]	Europe	CS	67.59	36	SSES (family/ friends)	S	IPAQ-SF	*	Fair
Smith et al., 2023 [46]	Australia	LS	61.7	1,984	5 items	S	Items (LTPA, MVPA)	+	Good
Wagner et al., 2020 [22]	Europe & Asia	CS	71.03	617	3 items	S	3 items on park-based PA	0	Good
Warner et al., 2011 [47]	Europe	LS	73.27	309	SSES-SF	S	1 item	+	Good
Wilcox et al., 2003 [27]	North America	CS	70.6	102	SSES	S	PASE	0	Good
Zhou et al., 2023 [65]	Asia	CS	66.6	523	PASSS	S	IPAQ-SF	*	Poor

Social support measures: *PASSS* Physical Activity Social Support Scale, *SSPAS* Social Support for Physical Activity Scale, *SSES* Social Support for Exercise Scale

Physical activity measures: *CHAMPS* Community Healthy Activities Model Program for Seniors, *GLTEQ* Godin’s Leisure Time Exercise Questionnaire, *IPAQ* International Physical Activity Questionnaire, *PASE* Physical Activity Scale for the Elderly

^a^*CS* Cross-sectional studies, *L* Longitudinal studies, *S* Subjective PA, *O* Objective PA

^b^*N* sample size

^c^*S* Subjective PA, *O* Objective PA

^d^ Results: 0 indicates no sig. relationship (*p* ≥ 0.05), + indicates sig. pos. relationship (*p* < 0.05), - indicates sig. neg. relationship, * indicates sig. mixed relationship

Of the studies reviewed in this category, 18 (72%) reported significant positive associations between SSPA and PA, while one study identified a significant negative relationship [55] and two studies reported both positive and negative associations [36, 56]. Notably, the two longitudinal studies reported significant positive associations between SSPA and PA, indicating that the former may have enduring effects on the latter – extending to six months [46] and even up to nine years later [45].

Of the four studies employing an objective measure for PA, two found significant positive associations between SSPA and PA [41, 42], while the other two did not yield significant results. One of these used an objective measure of SOSU [35], and the other combined three PA measures (an accelerometer and two self-report scales) to assess the relationship between TPA and SSPA [38].

Of the 23 studies that assessed PA subjectively, 14 (61%) used validated scales. Nine of these employed instruments designed specifically to measure PA in older adults: six used the PASE [23, 27, 55, 57–59] and three the CHAMPS [41, 42, 60]. The remaining seven used instruments designed for the general population: six used the IPAQ [36, 56, 61–64] and one the GLTEQ [65]. There appeared to be no clear consensus in this category regarding the relevance of the source of SOSU provision. Examining the sources of SOSU, Park et al. [55] reported a negative relationship between family SSPA and PA, whereas four other studies identified a significant positive association between family SOSU and PA [33, 36, 56, 65]. Findings related to support from friends were similarly mixed: Van Luchene et al. found a significant negative relationship between SSPA from friends and PA [36], although their study involved only 36 participants. Zhou et al. [56] found negative association between friends’ SOSU and LTPA. Meanwhile, a longitudinal study by Warner et al. demonstrated that baseline friends’ SOSU positively predicted exercise behaviour six months later [46].

In terms of gender-specific analysis, three studies focused exclusively on female participants [23, 24, 27], and two studies conducted separate analyses for men and women [29, 33]; all reported a statistically significant positive association between SSPA and PA. Sirotiak et al. discovered a significant positive relationship between SSPA and PA in rural older adults, but not in urban older adults [59].

### Emotional SOSU

In total, ten studies analysed the relationship between emotional SOSU and subjective PA (see Table 4). Of these, nine had a cross-sectional design [26, 34, 37, 39, 66–70] and one had a longitudinal design [32]. Six studies showed a significant positive relationship between emotional SOSU and PA, while three did not find a significant association.

**Table 4:** Overview of results: emotional social support and physical activity

Author, year	Continent	Study design ^a^	Mean age	*N* ^b^	SOSU measure	PA measure	Results ^c^	Quality
Komazawa et al., 2021 [33]	Asia	LS	68.9	3,911	2 items	1 item	0	Good
Krause et al., 1993 [67]	Asia	CS	68.7	1351	2 items	3 items	+	Good
Lee et al., 2025 [68]	North America	CS	69.26	13,771	4 items	1 item	0	Good
Loprinzi & Joyner, 2016 [35]	North America	CS	71.4	5,616	2 items	48 items	+	Good
Newsom et al., 2018 [71]	North America	CS	72.55	217	4 items	CHAMPS	+	Good
Watt et al., 2014 [69]	North America	CS	71.7	4,014	2 items	1 item	+	Good
Yamakita et al., 2015 [38]	Asia	CS	73.5	78,002	2 items	1 item	+	Good
Yuan et al., 2025 [70]	North America	CS	63.07	2,155	12 items (MIDUS)	2 items	+	Good
Zambrano Garza et al., 2024 [40]	North America	EMA	71.39	133	1 item	1 item/ accelerometer	+	Fair
Zimmer & McDonough, 2021 [60]	North America	CS	72.78	21,491	1 item (MOS)	PASE	0	Good

Social support measures: *MIDUS* MIDUS Social Support Scale, *MOS* Medical Outcomes Study Social Support Survey

Physical activity measures: *CHAMPS* Community Healthy Activities Model Program for Seniors, *PASE* Physical Activity Scale for the Elderly

^a^*CS* Cross-sectional studies, *L* Longitudinal studies, *EMA* Ecological momentary assessment design studies, *S* Subjective PA, *O* Objective PA

^b^*N* sample size

^c^ Results: 0 indicates no sig. relationship (*p* ≥ 0.05), + indicates sig. pos. relationship (*p* < 0.05), - indicates sig. neg. relationship, * indicates sig. mixed relationship

Yuan et al. [70] found inconclusive results regarding the association between emotional SOSU and various types of PA. They found that emotional SOSU from family was significantly negatively associated with moderate LTPA, whereas emotional SOSU from friends or partners was significantly positively associated with moderate LTPA. Emotional SOSU from friends is also significantly associated with vigorous LTPA. Loprinzi and Joyner also examined the relationship between emotional SOSU from various sources and PA [35]. Their findings indicate that emotional support, whether general or specifically provided by friends, was positively and significantly associated with PA among adults over 60. While emotional SOSU from any source was not significantly associated with PA solely among men, a significant relationship was observed between emotional SOSU from friends and PA among women. This suggests a gender-specific effect whereby friends’ support plays a more significant role in encouraging PA among older women.

### Informational SOSU

Two cross-sectional studies addressed the relationship between informational SOSU and PA, with one finding a significant positive association [60] and the other finding no significant association [70] (see Table 5).

**Table 5:** Overview of results: informational social support and physical activity

Author, year	Continent	Study design ^a^	Mean age	*N* ^b^	SOSU measure	PA measure	Results ^c^	Quality
Newsom et al., 2018 [71]	North America	CS	72.55	217	4 items	CHAMPS	+	Good
Zimmer & McDonough, 2021 [60]	North America	CS	72.78	21,491	1 item (MOS)	PASE	0	Good

Social support measures: *MOS* Medical Outcomes Study Social Support Survey

Physical activity measures: *CHAMPS* Community Healthy Activities Model Program for Seniors, *PASE* Physical Activity Scale for the Elderly

^a^*CS* Cross-sectional studies

^b^*N* Sample size

^c^ Results: 0 indicates no sig. relationship (*p* ≥ 0.05), + indicates sig. pos. relationship (*p* < 0.05), - indicates sig. neg. relationship

### Instrumental SOSU

Seven studies analysed the relationship between instrumental SOSU and PA (see Table 6). Six of these were cross-sectional [34, 37, 39, 70–72] and one was longitudinal [32]. The evidence for the association between instrumental SOSU and PA was inconclusive: four studies [34, 37, 39, 72] found a significant positive effect of instrumental SOSU on PA, whereas two [70, 71] found a significant negative effect. The longitudinal study did not report significant results [32]. The studies concerned varied considerably, however, in terms of both SOSU and PA measurements. For example, Loprinzi and Joyner operationalised instrumental SOSU with financial help [34], whereas Van Cauwenberg et al. only asked respondents about instrumental support from neighbours [72]. There was also considerable heterogeneity in PA outcomes across the studies: Yamakita et al. studied participation in sporting groups [37], whereas Perrino et al. and Van Cauwenberg et al. operationalised LPA involving walking [71, 72]. Zambarano Garza et al. [39], meanwhile, merged self-reported daily steps and objectively measured steps into one outcome. Furthermore, sample sizes varied from 217 [71] to 78,002 participants [37].

**Table 6:** Overview of results: instrumental social support and physical activity

Author, year	Continent	Study design ^a^	Mean age	*N* ^b^	SOSU measure	PA measure	Results ^c^	Quality
Komazawa et al., 2021 [33]	Asia	LS	68.9	3,911	2 items	1 item	0	Good
Loprinzi & Joyner, 2016 [35]	North America	CS	71.4	5,616	2 items	48 items	+	Good
Perrino et al., 2011 [72]	North America	CS	79.95	217	2 items	1 item on walking	-	Good
Van Cauwenberg et al., 2014 [75]	Europe	CS	74.3	50,986	1 item (TYPE	1 item on walking	+	Good
Yamakita et al., 2015 [38]	North America	CS	73.5	78,002	2 items	1 item	+	Good
Zambrano Garza et al., 2024 [40]	North America	EMA	71.39	133	1 item	1 item/ accelerometer	+	Fair
Zimmer & McDonough, 2021 [60]	North America	CS	72.78	21,491	1 item (MOS)	PASE	-	Good

Social support measures: *MOS* Medical Outcomes Study Social Support Survey

Physical activity measures: *PASE* Physical Activity Scale for the Elderly

^a^*CS* Cross-sectional studies, *L* Longitudinal studies, *EMA* Ecological momentary assessment design studies

^b^*N* Sample size

^c^ Results: 0 indicates no sig. relationship (*p* ≥ 0.05), + indicates sig. pos. relationship (*p* < 0.05), - indicates sig. neg. relationship

### Companionship SOSU

Six cross-sectional studies [28, 40, 70, 73–75] considered the effect of companionship SOSU on subjective PA (see Table 7). While the SOSU measures employed differed across these studies, five found a significant positive relationship between companionship SOSU and PA. Regarding the source of companionship SOSU Zambrano Garza et al. [40] found in a EMA study that PA with a romantic partner or a friend was associated with a higher daily step count and more MVPA, compared with activity undertaken alone or with other members of the social network. No such association was found, however, for other types of close social contact. It is noteworthy that this association reflected stable individual differences among romantic partners – that is, people who generally tended to exercise with their romantic partner were more physically active overall – rather than daily fluctuations within the same individual. With friends, both patterns were evident: people who generally exercised with a friend were more physically active overall reported more minutes of MVPA on the days when they were specifically active with a friend.

**Table 7:** Overview of results: companionship social support and physical activity

Author, year	Continent	Study design ^a^	Mean age	*N* ^b^	SOSU measure	PA measure	Results ^c^	Quality
Ory et al., 2016 [75]	North America	CS	69	272	2 items	4 items	+	Good
Salvador et al., 2009 [28]	South America	CS	NR	152	NEWS	IPAQ	+	Good
Shores et al., 2009 [76]	North America	CS	74.28	454	1 item	7-Day PA recall questionnaire	+	Good
Wendt Böhm et al., 2016 [73]	South America	CS	70.07	1,285	PASSS	IPAQ (leisure domain)	+	Good
Zambrano Garza et al., 2025 [41]	North America	EMA	71.09	139	1 item	1 item, accelerometer	+	Fair
Zimmer & McDonough, 2021 [60]	North America	CS	72.78	21,491	1 item (MOS)	PASE	0	Good

Social support measures: *NEWS* Neighborhood Environment Walkability Scale, *PASSS* Physical Activity Social Support Scale, *MOS* Medical Outcomes Study Social Support Survey

Physical activity measures: *IPAQ* International Physical Activity Questionnaire, *PASE* Physical Activity Scale for the Elderly

^a^*CS* Cross-sectional studies, *EMA* Ecological momentary assessment design studies

^b^*N* Sample size

^c^ Results: 0 indicates no sig. relationship (*p* ≥ 0.05), + indicates sig. pos. relationship (*p* < 0.05), - indicates sig. neg. relationship

### Type of PA measurement and specific PA categories

A substantial proportion of the studies (*n* = 49, 90%) relied on self-reported measures of PA, which may have introduced bias due to participants’ tendency to overestimate their activity levels [76]. Seven studies [35, 38–43] also collected objective PA data using accelerometers; however, this method can also introduce bias, as participants may increase their activity simply because they are being monitored [76]. Three studies [35, 41, 43] employed only objective measures. Of these, two found significant positive associations between SOSU and PA – specifically with MVPA in Carlson et al. [41] and with LPA in Chen et al. [43]. Three studies combined subjective and objective measures into a single metric. Two of these found no significant association [38, 39], while one found a significant positive association [40]. Another study assessed subjective and objective PA separately and found significant positive associations for each [42].

For the subjective measurement of PA, twelve studies employed tools specifically designed for older adults. Three studies used the Community Healthy Activities Model Program for Seniors questionnaire (CHAMPS) [41, 42, 60], eight used the Physical Activity Scale for the Elderly (PASE) [23, 26, 27, 55, 57–59, 70] and one used the Older Adult Exercise Status Inventory [25]. The findings of these studies were mixed. Several reported a positive association between at least one type of SOSU and PA [23, 24, 41, 42, 57, 59, 60], while others found no significant association [27, 58]. Notably, three studies reported negative associations, although they each focused on a different type of SOSU: Marthammuthu examined general SOSU [26], Park focused on SSPA [55] and Zimmer and McDonough assessed instrumental SOSU [70].

Variation was also evident in the type of PA assessed, namely moderate-to-vigorous PA (MVPA), leisure-time PA (LTPA), light PA (LPA) and total PA (TPA). Eight studies specifically focused on walking, a form of LPA, with six reporting a significant positive association between this form of activity and SOSU [29, 41, 42, 61, 72, 74] and one identifying a significant negative association [71]. This inconsistency may stem from the different methods used to measure walking: three studies relied on a single-item question [71, 72, 74], while others employed validated instruments such as the Community Healthy Activities Model Program for Seniors questionnaire [41, 42] or the International Physical Activity Questionnaire [71]. One study used an accelerometer, finding a significant association between general SOSU and LPA but no association between the former and MVPA [43].

The relationship between SOSU and LTPA (which includes walking for leisure) appeared particularly strong. Of 16 studies examining LTPA, 13 (80%) reported a positive association [24, 31, 33, 41, 42, 44, 49, 51, 56, 61, 65, 69, 77], while three found no significant relationship [27, 62, 67]. MVPA was the most frequently assessed PA outcome across studies, but findings in this category were more mixed.

### Gender

We found evidence for gender-related differences in the association between SOSU and PA. Of the five studies that examined women only, three reported a significant positive relationship between SOSU and PA [23–25], one found a significant negative relationship [26] and one found no significant association [27]. Seven studies differentiated their results by gender [29–35], of which five found significant associations between SOSU and PA. Across the studies that reported gender-stratified results, the association between SOSU and PA tended to be stronger in women. Lian et al. found significant positive associations for both men and women, but the relationship was more pronounced in women [33]. Blakoe et al. and Kaplan et al. observed significant associations only in women, with no corresponding effect in men [30, 31]. Similarly, Loprinzi and Joyner reported positive associations between emotional SOSU and PA for the full sample and for women but not for men; for men alone, the only significant positive association observed was between instrumental (financial) SOSU and PA [34].

### Sources of SOSU

When we examine the sources of functional SOSU across different SOSU types, the findings remain somewhat inconclusive. Most studies investigating SOSU from family members reported a significant positive association with PA [23, 33, 56, 57, 61, 65, 73]. However, three studies found no significant relationship [25, 52, 62], and two identified a negative association [55, 67]. The evidence regarding SOSU from friends was even less consistent. De Sousa et al. reported no significant association [62], while Van Luchene et al. and Zhou et al. found a negative relationship [36, 56]; in contrast, six other studies identified a positive association between SOSU from friends and PA [25, 40, 46, 57, 61, 69, 73]. A ten-day daily diary study found that companionship SOSU from a friend or partner was significantly positively associated with daily minutes of MVPA and daily step counts [40]. Notably, two longitudinal studies found that SOSU from friends at baseline was significantly related to PA at follow-up, indicating a consistent association over time [25, 46]. Interestingly, Corseuil Giehl et al. found that friendship-based SOSU was significantly associated only with walking for more than 150 min per week, whereas family SOSU was linked to walking for both 10–149 min and more than 150 min per week [61]. Additionally, Loprinzi and Joyner analysed their sample by age group (general (60 + years), younger (60–69 years) and older (70 + years)) and found that SOSU from a spouse was significantly associated with PA only in the older age group [34].

#### Results of the meta-analysis

The second research question concerns the overall magnitude of the association between functional SOSU, with particular focus on the variations in the association by PA type. This is particularly important, as the results of the systematic review revealed inconclusive results regarding both sources of SOSU and PA measures. Furthermore, there exists substantial heterogeneity across the studies in terms of PA outcomes and different SOSU types, including their measurement (e.g. through single-item questions versus validated scales). This variability limits the comparability of findings across studies, which is why the meta-analysis was restricted to studies assessing general SOSU or SSPA and employed subjective PA measures. Of the 54 articles included in the systematic review, 33 (61%) were thus eligible for inclusion in the meta-analysis. Of these, 13 (39%) were conducted in Asia, twelve (36%) in North America, six (18%) in Europe, one (4%) in South America and one (4%) in both Europe and Asia. The sample sizes varied from 36 [36] to 19,298 [48]. Out of the 33 studies, three employed a longitudinal design [25, 44, 46], while 30 were cross-sectional.

**Fig. 2:**
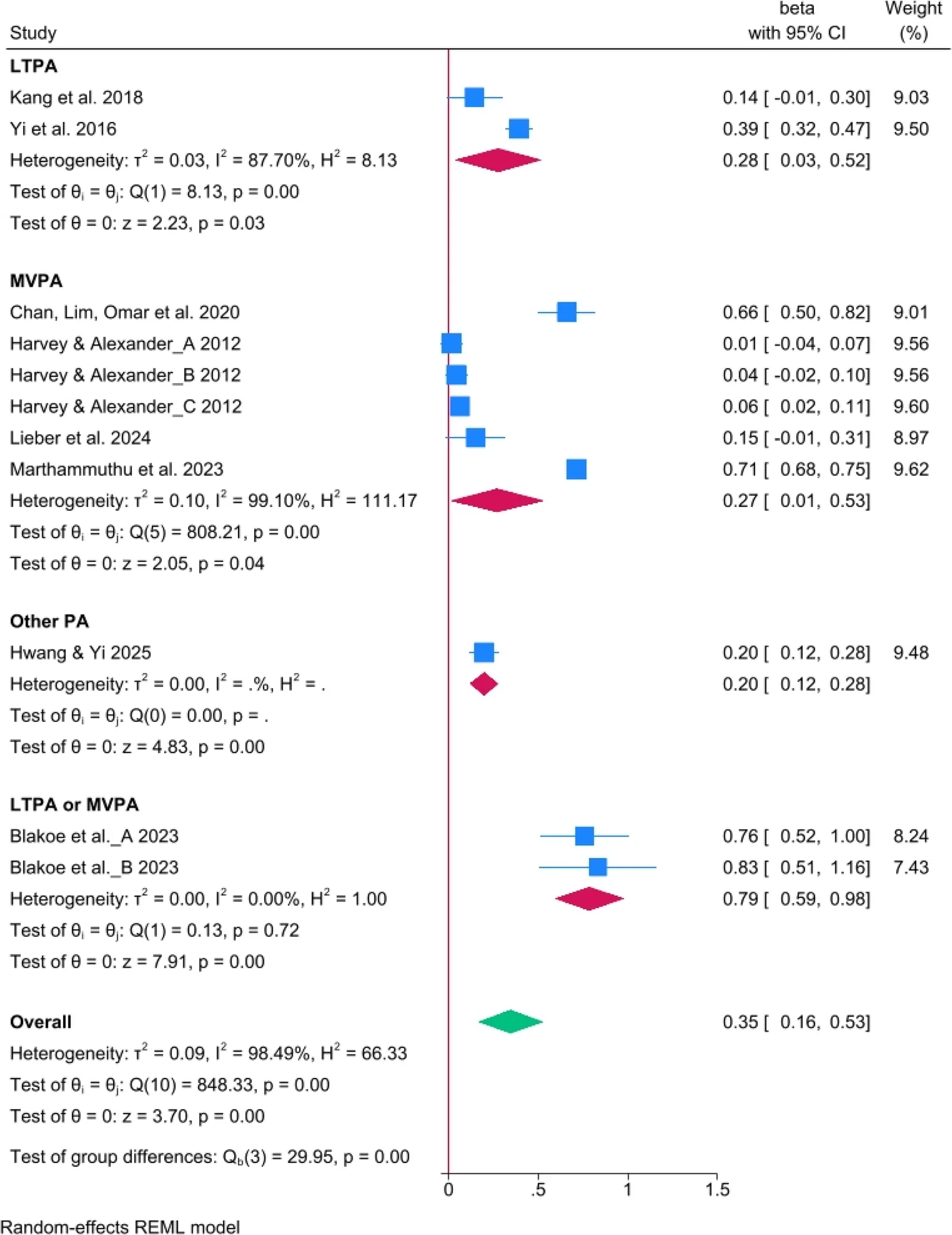
Forest plot for general social support with continuous physical activity outcomes

**Fig. 3:**
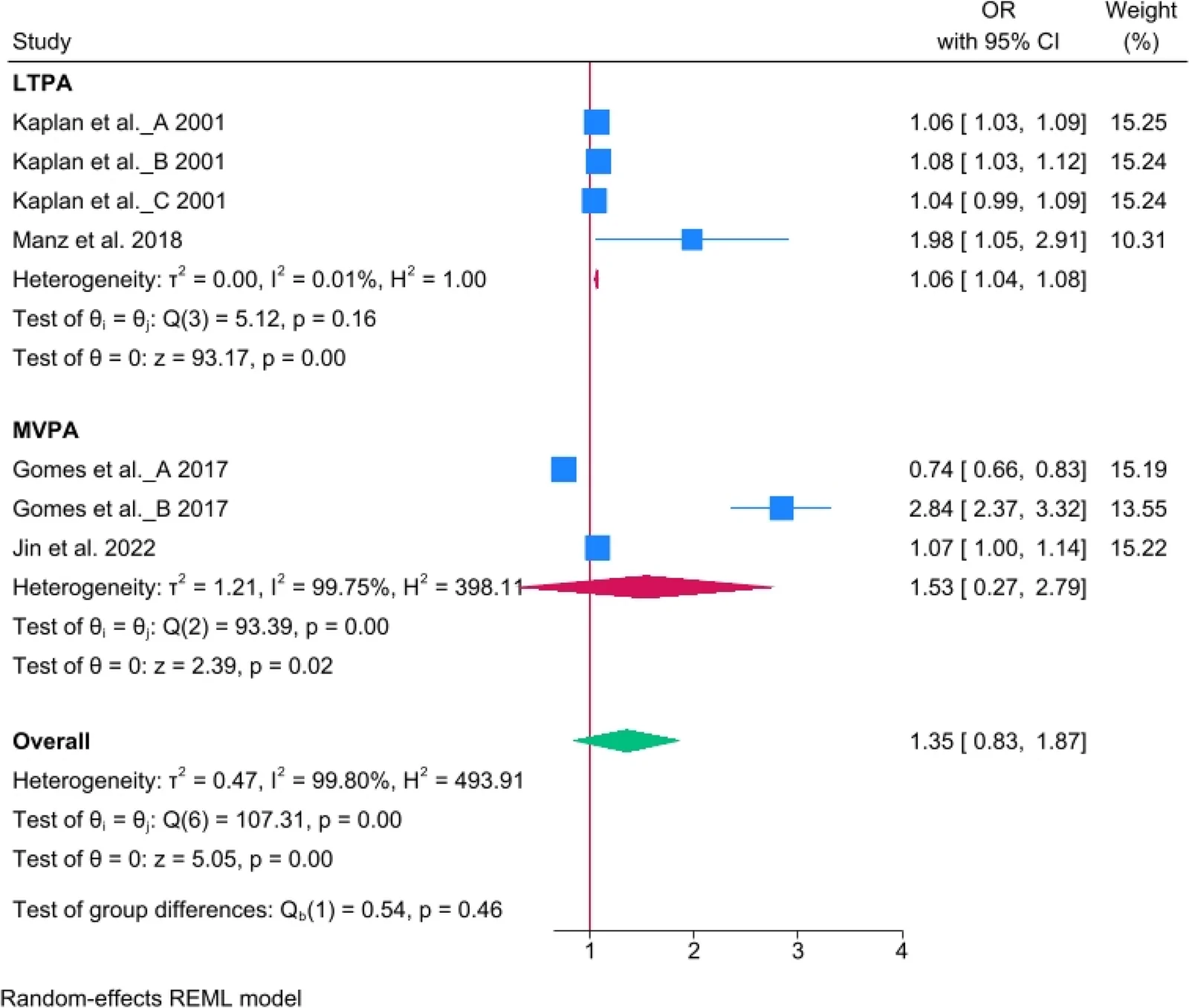
Forest plot for general social support with binary physical activity outcomes

**Fig. 4:**
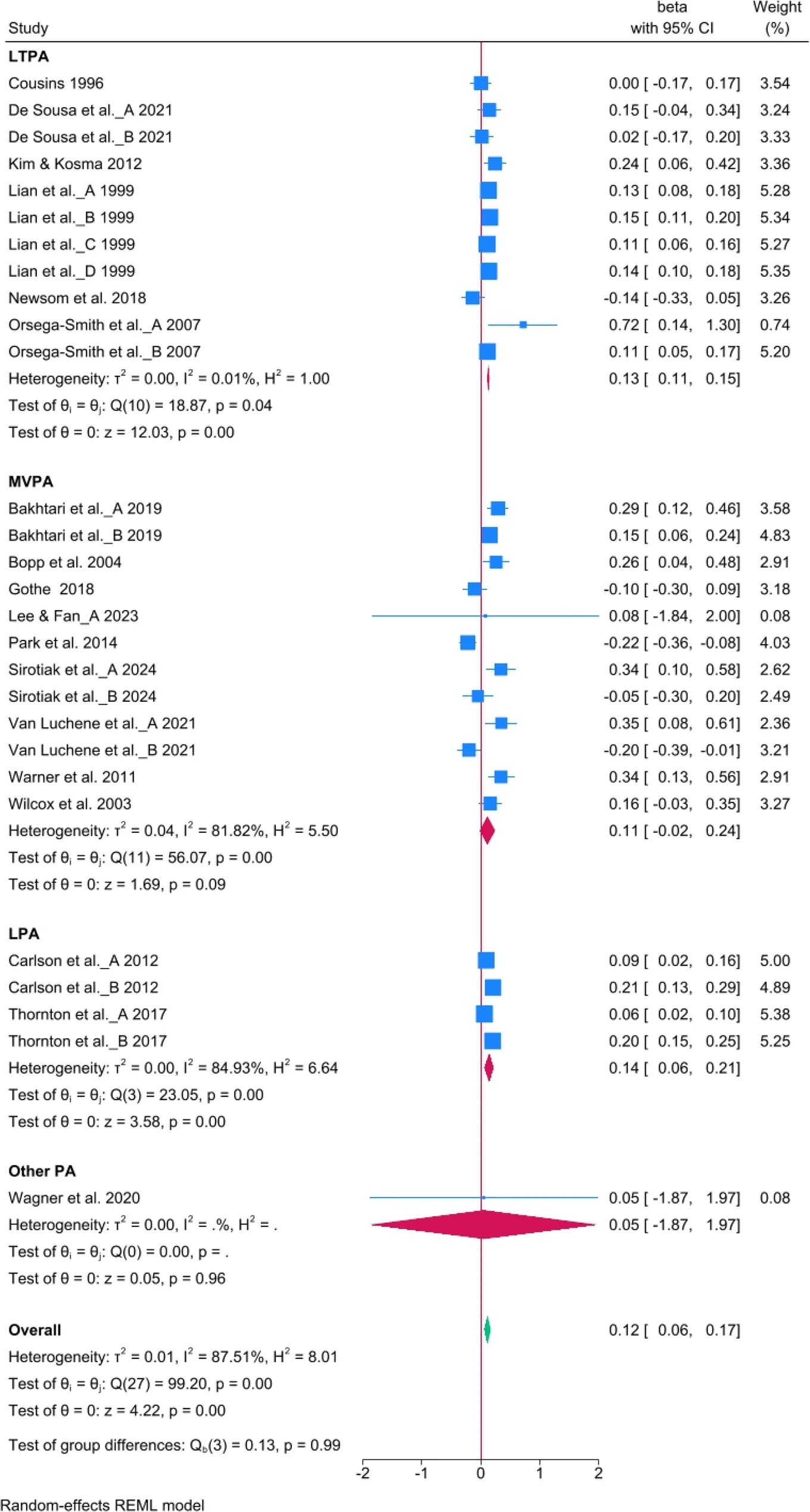
Forest plot for social support for physical activity with continuous physical activity outcomes

**Fig. 5:**
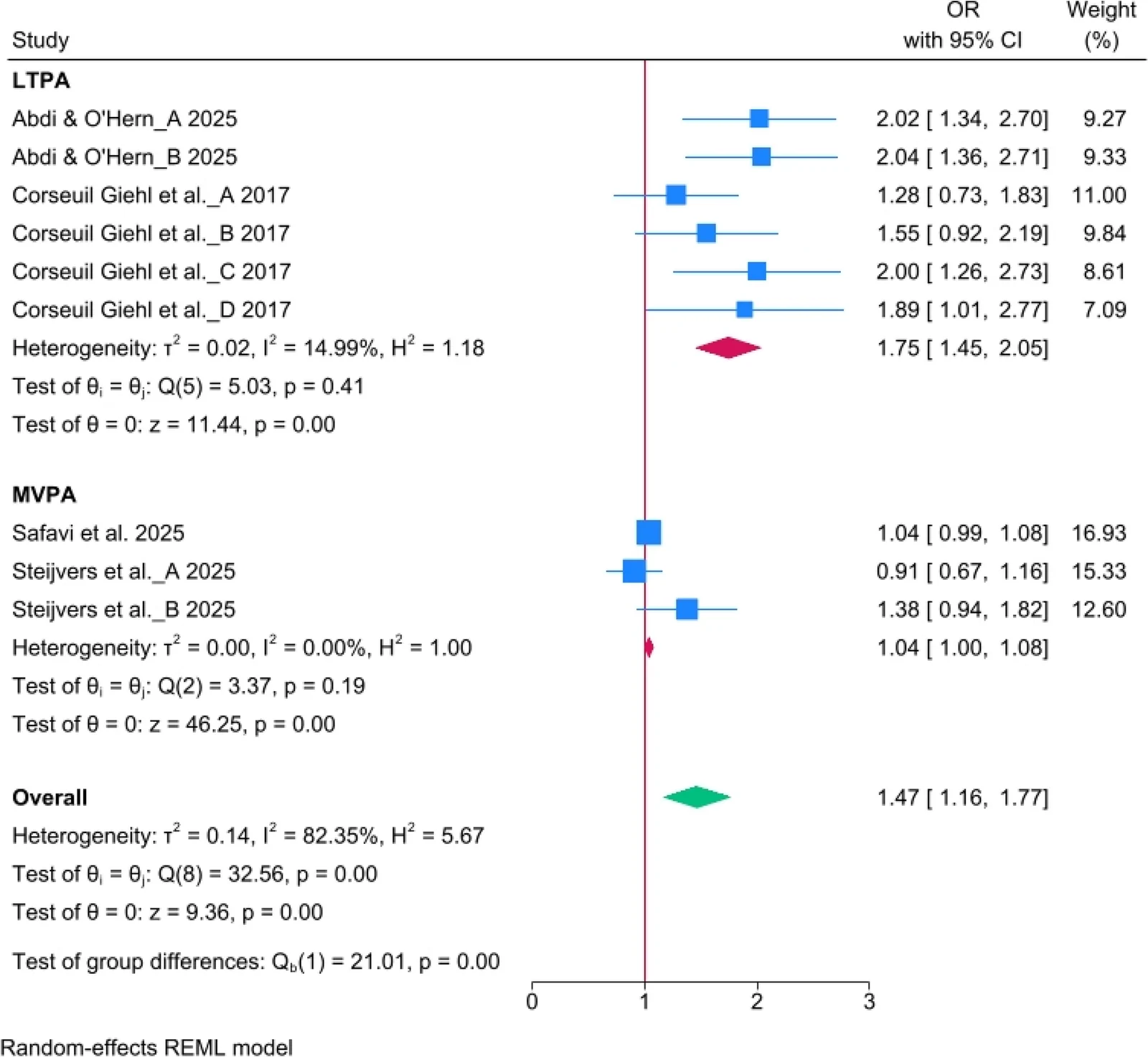
Forest plot for social support for physical activity with binary physical activity outcomes

We generated four separate forest plots: for general SOSU and SSPA, each for continuous (Figs. 2 and 4) and binary PA outcomes (Figs. 3 and 5) In addition, meta-regressions were performed separately for continuous and binary PA, including 39 and 16 models, respectively (Tables 12 and 13). An overview of the studies included in the meta-analysis is presented in Tables 8, 9, 10 and 11, covering general SOSU and SSPA with continuous and binary PA outcomes, respectively.

**Table 8:** Overview of studies included in the meta-analysis for general social support with continuous physical activity

Author, year	Continent	Study design ^a^	Mean age	*N* ^b^	Sub-sample	SOSU measure	PA measure	Quality
Blakoe et al._A, 2023 [31]	Europe	CS	74.9	2850	men	DSSI	MVPA (2 items): doing PA in the leisure time at least several times a week, reported frequent PA during daily activity	Good
Blakoe et al._B, 2023 [31]	Europe	CS	76.8	4173	women	DSSI	MVPA (2 items): doing PA in the leisure time at least several times a week, reported frequent PA during daily activity	Good
Chan et al., 2020 [48]	Asia	CS	67.34	3969		DSSI	GPAQ	Good
Harvey & Alexander_A, 2012 [26]	North America	L	69	671	women	6 items	3 items	Good
Harvey & Alexander_B, 2012 [26]	North America	L	69	671	women	6 items	3 items	Good
Harvey & Alexander_C, 2012 [26]	North America	L	69	671	women	6 items	3 items	Good
Hwang & Yi, 2025 [50]	Asia	CS	60.24	974		MSPSSS	GLTEQ (3 items)	Good
Kang et al., 2018 [52]	Asia	CS	69.28	332		SSQ	GLTEQ	Good
Lieber et al. 2024 [53]	North America	CS	70.1	178		ISEL-12	GPAQ	Fair
Marthammuthu et al., 2023 [27]	Asia	CS	70.83	1221	women	DSSI	PASE	Good
Yi et al., 2016 [54]	Asia	CS	67.11	1580		5 items	3 items: assessing exercise frequency and duration	Good

Social support measures: *DSSI* Duke Social Support Index, *ISEL-12* Interpersonal Support Evaluation List, *MSPSS* Multidimensional Scale of Perceived Social Support, *SSQ* Perceived Social Support Questionnaire

Physical activity measures: *GLTEQ* Godin’s Leisure Time Exercise Questionnaire, *GPAQ* Global Physical Activity Questionnaire, *PASE* Physical Activity Scale for the Elderly

^a^*CS* Cross-sectional studies, *L* Longitudinal studies

^b^*N* Sample size

**Table 9:** Overview of studies included in the meta-analysis for general social support with binary physical activity

Author, year	Continent	Study design ^a^	Mean age	*N* ^a^	Sub-sample	SOSU measure	PA measure	Quality
Gomes et al._A, 2017 [49]	Europe	CS	67.8	19,298		1 item: received help in the last 12 months	2 items: PA frequency	Good
Gomes et al._B, 2017 [49]	Europe	CS	67.8	19,298		1 item: given help in the last 12 months	2 items: PA frequency	Good
Jin et al., 2022 [51]	Asia	CS	69	778		SSRS	IPAQ-SF	Good
Kaplan et al._A, 2001 [32]	North America	CS	72.25	12,611		4 items: Someone they could confine in; someone they could count on; someone who could give them advice; someone who made them feel loved	1 item: Monthly LTPA more than 15 min	Good
Kaplan et al._B, 2001 [32]	North America	CS	72.25	7,723	women	4 items: Someone they could confine in; someone they could count on; someone who could give them advice; someone who made them feel loved	1 item: Monthly LTPA more than 15 min	Good
Kaplan et al._C, 2001 [32]	North America	CS	72.25	4,888	Men	4 items: Someone they could confine in; someone they could count on; someone who could give them advice; someone who made them feel loved	1 item: Monthly LTPA more than 15 min	Good
Manz et al., 2018 [45]	Europe	L	60	1,143		1 item (from Oslo-3 Social Support Scale)	1 item: Frequency of LTPA	Good

Social support measures: *SSRS* Social Support Rate Scale

Physical activity measures: *IPAQ* International Physical Activity Questionnaire

^a^*CS* Cross-sectional studies, *L* Longitudinal studies

^b^*N* Sample size

We estimated pooled effects using a random-effects model (REML) to account for between-study variance. Heterogeneity was extremely high for the continuous (general SOSU *I*^2^ = 98.49%, SSPA *I*^2^ = 87.51%) and binary outcome (general SOSU *I*^2^ = 99.80%, SSPA *I*^2^ = 82.35%), indicating that much of the variability in effect sizes reflects true differences between studies. Even though this aligns with the heterogeneity commonly found in meta-analyses [78], pooled effect estimates should be interpreted with caution. Leave-one-out sensitivity analyses showed that the pooled effect remained stable when omitting individual studies, suggesting that no single study disproportionately influenced the overall estimate. This was true for both the continuous PA outcome (theta = 0.19, 95% CI: 0.11–0.26) and the binary PA outcome (theta = 1.42, 95% CI: 1.12–1.70).

### General SOSU

The forest plot in Fig. 2 summarises the standardised beta coefficients across eleven models for general SOSU with a continuous PA outcome. The pooled effect size of β = 0.35 (95% CI: 0.16–0.53) indicates a moderate, statistically significant positive association between general SOSU and PA. This suggests that studies measuring general SOSU tend to report meaningful positive associations with continuous PA outcomes.

The forest plot in Fig. 3 summarises the odds ratios across the seven models for general SOSU with a binary PA outcome. The pooled OR of 1.35 (95% CI: 0.83–1.87) indicates a small positive but non-significant association. Effect sizes were inconsistent across models, with one model reporting a negative association. This model measured received SOSU (Gomes et al._A 2017), whereas another model from the same study examined given SOSU (Gomes et al._B 2017) found a positive association, suggesting that the direction of SOSU flow may matter.

### SSPA

The forest plot in Fig. 4 presents the standardised beta coefficients from 28 models examining the association between SSPA and a continuous PA outcome. The pooled effect size of β = 0.12 (95% CI: 0.06–0.17) indicates a small but statistically significant positive association. The result remains stable when individual studies are excluded, indicating the robustness of the overall result despite the considerable heterogeneity in effect size. Furthermore, when the specific PA categories were analysed separately, the meta-analysis revealed significant positive associations between SSPA and each type of PA.

The forest plot in Fig. 5 summarises the odds ratios across the nine models for SSPA with a binary PA outcome. The pooled effect size of OR = 1.47 (95% CI: 1.16–1.77) indicates a small-to-moderate positive association between SSPA and PA in older adults. When the specific PA categories were analysed separately, the meta-analysis revealed a significant moderate positive association for LTPA (OR = 1.75, 95% CI: 1.45–2.05), but no significant association for MVPA (OR = 1.04, 95% CI: 1.00–1.08).

#### Meta-regression analysis

The forest plots suggested extreme heterogeneity, which is why meta-regressions were conducted to explore potential sources of variability. Table 12 presents the results of the multivariate meta-regression, where all variables were regressed on the effect size for the continuous PA outcome. Each coefficient reflects the association between a given study characteristic and effect size, adjusted for all other variables in the model. For SOSU measures, results suggest that general SOSU is associated with a significantly increased effect size compared to studies using SSPA (see Table 12, Model 1). We therefore adjusted the meta-regression models for type of SOSU where feasible. According to the results, sample characteristics (gender and age distribution) and number of control variables are significant yet marginally associated with the effect size between SOSU and PA. Effect sizes also tended to be somewhat stronger in Europe and Asia than in North America and were smaller in longitudinal than in cross-sectional study designs. Source of support (general, family, friends) was not significantly associated with the effect size.

**Table 10:** Overview of studies included in the meta-analysis for social support for physical activity with continuous physical activity outcomes

Author, year	Continent	Study design ^a^	Mean age	*N* ^b^	Sub-sample	SOSU measure	PA measure	Quality
Bakhtari et al._A, 2019 [58]	Asia	CS	69.17	340		SSES (family)	PASE-SF	Good
Bakhtari et al._B, 2019 [58]	Asia	CS	69.17	340		SSES (friends)	PASE-SF	Good
Bopp et al., 2004 [24]	North America	CS	70.6	102	women	SSES (family)	PASE (strength training)	Good
Cousins, 1996 [25]	North America	CS	76.7	136	women	4 items	Exercise level last 7 days	Fair
Carlson et al._A, 2012 [42]	North America	CS	74.4	707		SSES (adapted, 4 items)	CHAMPS (walking for transportation)	Good
Carlson et al._B, 2012 [42]	North America	CS	74.4	709		SSES (adapted, 4 items)	CHAMPS (walking for leisure)	Good
de Sousa et al., 2021_A [78]	South America	CS	69.9	208		SSPAS (friends)	IPAQ (leisure domain)	Good
de Sousa et al., 2021_B [78]	South America	CS	69.9	208		SSPAS (family)	IPAQ (leisure domain)	Good
Gothe, 2018 [39]	North America	CS	64.8	110		SSES	Score: Accelerometer (7 consecutive days), PASE, GLTEQ	Good
Kim & Kosma, 2013 [65]	Asia	CS	68.56	290		SSES (family)	GLTEQ	Good
Lee & Fan, 2023 [61]	Asia	CS	71.41	183		5 items: Assessing SOSU from family and friends for PA	PASE	Good
Lian et al., 1999_A [34]	Asia	CS	68.69	1052	men	1 item: Family encouragement to exercise	Self-reported weekly frequency of MVPA	Good
Lian et al., 1999_B [34]	Asia	CS	68.57	1442	women	1 item: Family encouragement to exercise	Self-reported weekly frequency of MVPA	Good
Lian et al., 1999_C [34]	Asia	CS	68.69	1052	men	1 item: Exercising among family members	Self-reported weekly frequency of MVPA	Good
Lian et al., 1999_D [34]	Asia	CS	68.57	1442	women	1 item: Exercising among family members	Self-reported weekly frequency of MVPA	Good
Newsom et al., 2018 [71]	North America	CS	72.55	217		4 items	CHAMPS	Good
Orsega-Smith et al., 2007_A [62]	North America	CS	67.7	1579		SSES (family)	2 items: type of LTPA + frequency over the last week	Good
Orsega-Smith et al., 2007_B [62]	North America	CS	67.7	1579		SSES (friends)	2 items: type of LTPA + frequency over the last week	Good
Park et al., 2014 [56]	Asia	CS	71.62	187		SSES (family)	PASE	Good
Sirotiak et al., 2024_A [66]	North America	CS	69.49	47	rural	SSES	PASE	Fair
Sirotiak et al., 2024_B [66]	North America	CS	67.63	48	urban	SSES	PASE	Fair
Thornton et al., 2017_A [43]	North America	CS	74.4	726		8 items: How often during the past 6 months their family, friends, acquaintances or co-workers (1) walked or exercised with them, (2) gave them encouragement to do physical activity, (3) made positive comments about the participant’s physical appearance, and (4) criticized or made fun of them for walking or exercising	CHAMPS (walking domain: walking for errands)	Good
Thornton et al., 2017_B [43]	North America	CS	74.4	726		8 items: How often during the past 6 months their family, friends, acquaintances or co-workers (1) walked or exercised with them, (2) gave them encouragement to do physical activity, (3) made positive comments about the participant’s physical appearance, and (4) criticized or made fun of them for walking or exercising	CHAMPS (walking domain: walking for leisure/ exercise)	Good
van Luchene et al., 2021_A [37]	Europe	CS	67.59	36		SSES (family)	IPAQ-SF	Fair
van Luchene et al., 2021_B [37]	Europe	CS	67.59	36		SSES (friends)	IPAQ-SF	Fair
Wagner et al., 2020 [22]	Europe & Asia	CS	71.03	526		3 items	3 items on park-based PA	Good
Warner et al., 2011 [47]	Europe	L	73.27	309		SSES-SF	1 item	Good
Wilcox et al., 2003 [27]	North America	CS	70.6	102	women	SSES	PASE	Good

Social support measures: *SSPAS* Social Support for Physical Activity Scale, *SSES* Social Support for Exercise Scale

Physical activity measures: *CHAMPS* Community Healthy Activities Model Program for Seniors, *GLTEQ* Godin’s Leisure Time Exercise Questionnaire, *IPAQ* International Physical Activity Questionnaire, *PASE* Physical Activity Scale for the Elderly

^a^*CS* Cross-sectional studies, *L* Longitudinal studies

^b^ N = sample size

**Table 11:** Overview of studies included in the meta-analysis for social support for physical activity with binary physical activity outcomes

Author, year	Continent	Study design ^a^	Mean age	*N* ^b^	Sub-sample	SOSU measure	PA measure	Quality
Abdi & O’Hern, 2025_A [30]	Asia	CS	60	2064	Men	1 item	1 item	Good
Abdi & O’Hern, 2025_B [30]	Asia	CS	60	1628	Women	1 item	1 item	Good
Corseuil Giehl et al._2017_A [77]	South America	CS	70.3	1,705		SSES (friends & neighbours)	IPAQ walking (10–149 min/week)	Good
Corseuil Giehl et al., 2017_B [77]	South America	CS	70.3	1,705		SSES (friends & neighbours)	IPAQ walking (> 150 min/week)	Good
Corseuil Giehl et al., 2017_C [77]	South America	CS	70.3	1,705		SSES (family)	IPAQ walking (10–149 min/week)	Good
Corseuil Giehl et al., 2017_D [77]	South America	CS	70.3	1,705		SSES (family)	IPAQ walking (> 150 min/week)	Good
Safavi et al., 2025 [63]	Asia	CS	65.89	550		SSES	IPAQ-SF	Good
Steijvers et al., 2025_A [64]	Europe	CS	68	1,975		1 item	IPAQ-SF (LPA)	Good
Steijvers et al., 2025_B [64]	Europe	CS	68	1,975		1 item	IPAQ-SF (MVPA)	Good

Social support measures: *SSES* Social Support for Exercise Scale

Physical activity measures: *IPAQ* International Physical Activity Questionnaire

^a^*CS* Cross-sectional studies, *L* Longitudinal studies

^b^*N* Sample size

The results of the meta-regression analysis on PA as a binary outcome are shown in Table 13. Similar to the results in the meta-regression of the continuous outcome variable, also when measuring PA as binary outcome variable, effect sizes are significantly larger in studies that use general SOSU, compared to SSPA. Effect sizes were also significantly larger in models that measures MVPA, compared to LTPA, and for studies conducted in South America or Europe compared with North America. Contrary to the results in the meta-regression of the continuous outcome variable, the number of control variables and publication year are significantly, but negatively associated with the effect size when measuring PA as binary outcome variable, while the gender proportion and sample mean age are not significantly associated with the effect size.

#### Asymmetry and publication bias

**Fig. 6:**
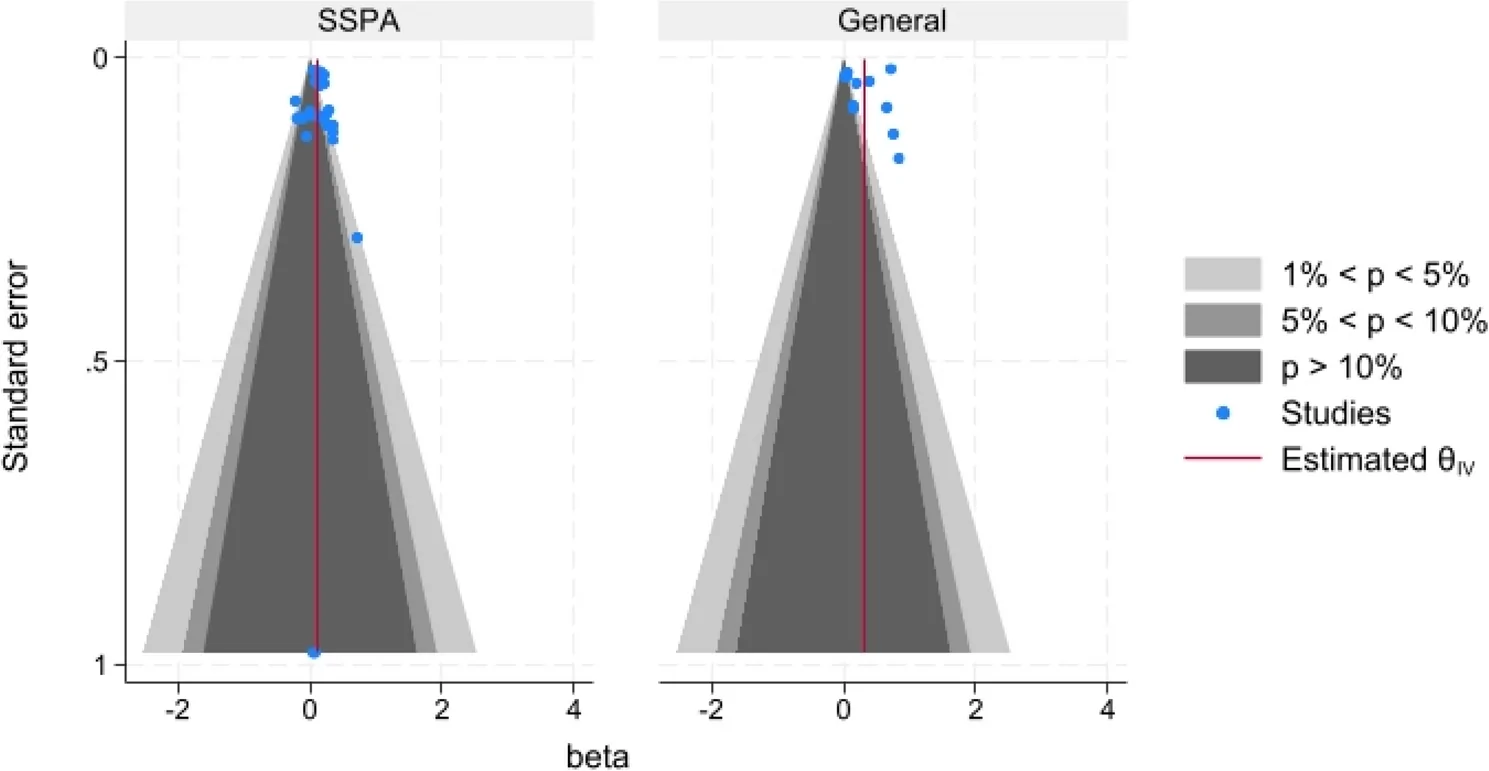
Funnel plot for social support for physical activity with continuous physical activity outcomes

We examined asymmetry using funnel plots, which show the distribution of the 39 studies included in the meta-regression, with effect sizes plotted against their standard errors [13]. Figure 6 indicates some risk of publication bias towards an overestimation of the effect size; this bias was higher in studies using general SOSU than in those using SSPA.

**Fig. 7:**
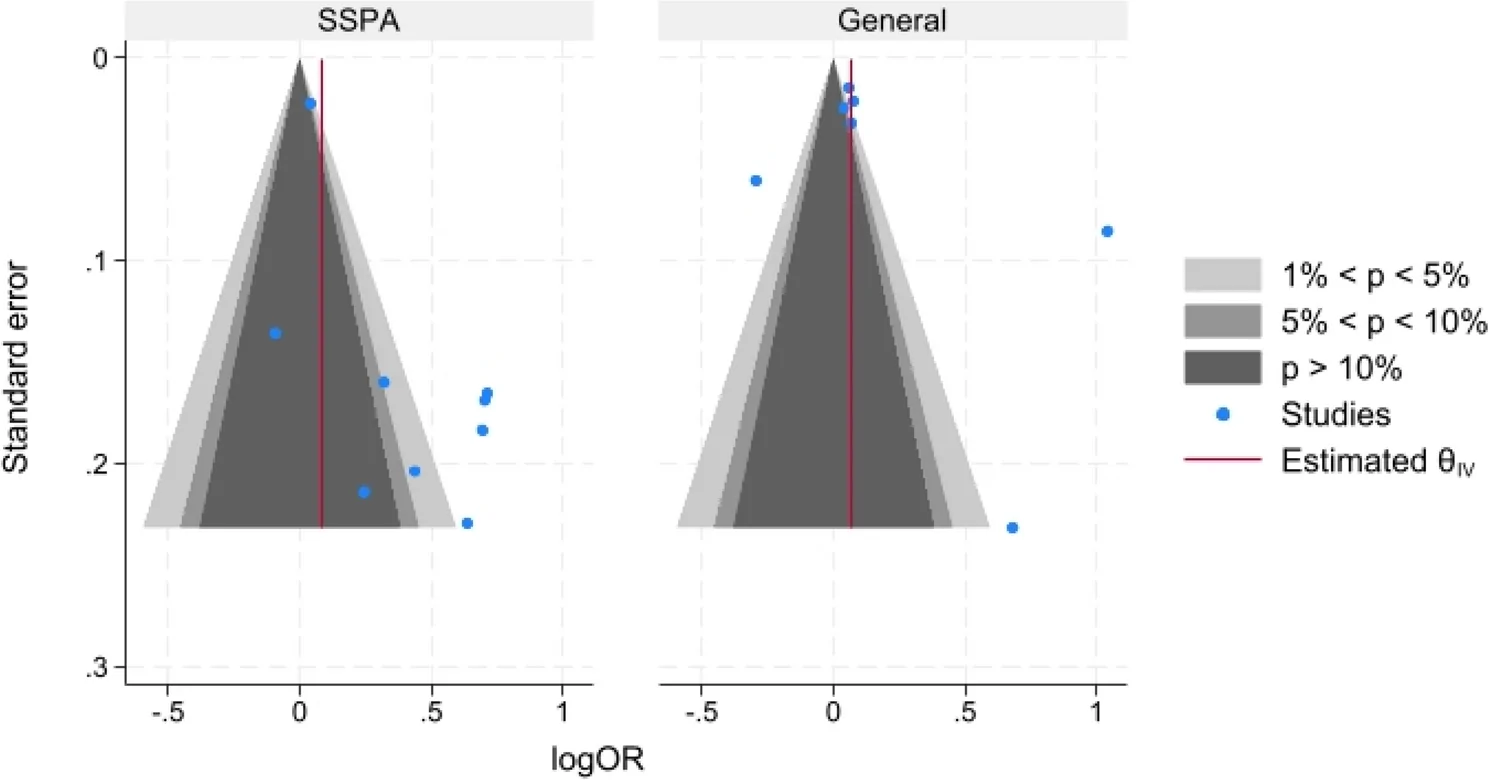
Funnel plot for social support for physical activity with binary physical activity outcomes

Figure 7 presents the funnel plots for the binary PA outcomes, with effect sizes expressed as log odds ratios (log OR). Although most studies fall within the funnel plot contours, publication bias is difficult to assess reliably when fewer than 10 studies are included [79]. Therefore, the plots should be interpreted with caution.

**Table 12:** Meta-regression of SOSU on PA continuous

	beta	SE
*Model 1* ^*a*^		
Type of SOSU measure		
SSPA (*ref*.)		
General SOSU	0.284^**^	(0.092)
Type of PA		
LTPA (*ref*.)		
MVPA	0.101	(0.0725)
LPA	0.429^**^	(0.147)
Other PA	-0.104	(0.170)
LTPA or MVPA	0.571^***^	(0.149)
*Model 2* ^b^		
Support source		
General (*ref*.)		
Family	0.063	(0.121)
Friends	-0.011	(0.144)
% male	0.003^*^	(0.001)
Mean age	0.005^***^	(0.001)
Year	0.000^***^	(0.000)
Design		
Cross-sectional (*ref*.)		
Longitudinal	-0.495^**^	(0.185)
*Model 3* ^c^		
Region		
North America (*ref.*)		
South America	0.083	(0.147)
Europe	0.302^**^	(0.102)
Asia	0.155^**^	(0.059)
*Model 4* ^d^		
No. control variables	0.027^***^	(0.003)
*N*(models)	39	

^a^*Model 1* adjusted for nestedness of models in studies using dummy variables. ^b^*Model 2* adjusted for nestedness of models in studies using dummy variables and type of SOSU measure. Reduced model with fewer adjustments where convergence failed: ^c^*Model 3* adjusted for type of SOSU measure; ^d^*Model 4* unadjusted. Estimates for adjustment variables and constants not shown. Standardised beta coefficients with standard errors in parentheses

^*^p < 0.05, ^**^p < 0.01, ^***^p < 0.001

**Table 13:** Meta-regression of SOSU on PA binary

	OR	CI
*Model 1* ^a^		
Type of SOSU measure		
SSPA (*ref*.)		
General SOSU	1.706^***^	(0.895, 2.516)
*Model 2* ^b^		
% male	-0.000	(-0.013, 0.012)
Mean age	-0.080	(-7.717, 7.557)
No. control variables	0.087^**^	(0.025, 0.150)
Year	0.001^**^	(0.000, 0.001)
Region		
North America (*ref.*)		
South America	1.656^***^	(1.006, 2.306)
Europe	1.133^**^	(0.318, 1.948)
Asia	1.040	(-0.059, 2.139)
Type of PA		
LTPA (*ref*.)		
MVPA	1.133^**^	(0.318, 1.948)
*N*(models)	16	

^a^*Model 1* adjusted for nestedness of models in studies using dummy variables. ^b^*Model 2* adjusted for nestedness of models in studies using dummy variables and type of SOSU measure. Estimates for adjustment variables and constants not shown. Odds ratios with 95% confidence intervals in parentheses

^*^p < 0.05, ^**^p < 0.01, ^***^p < 0.001

Small-study effects were assessed using Egger’s test. For the continuous PA outcome, the test was not statistically significant (β = 0.30, SE = 0.57, z = 1.04, *p* = 0.299), indicating no evidence of funnel plot asymmetry. Egger’s test within study subgroups (PA type and SOSU measure) also did not reveal significant asymmetry. In contrast, for the binary PA outcome, Egger’s test was statistically significant (β = 2.71, SE = 0.630, z = 4.30, *p* < 0.0000), suggesting that smaller studies may overestimate the effect size. In our dataset, this appears to apply to the study by Manz et al. [44], which had a comparatively small sample, large effect, and large confidence interval. Excluding this study reduced the pooled effect slightly (OR = 1.39, 95% CI: 1.11–1.68) compared with the pooled estimate including all studies (OR = 1.42, 95% CI: 1.14–1.70). Overall, while substantial heterogeneity exists, sensitivity analyses and assessment of small-study effects suggest that the pooled estimates are robust, though the binary PA outcome may be slightly influenced by smaller studies.

## Discussion

The primary aim of this systematic review was to thoroughly collect and systematise the evidence for the relationship between functional SOSU and PA in community-dwelling older adults. In addition, the meta-analysis aimed to quantify the evidence regarding general SOSU and subjective PA, as well as SSPA and subjective PA. We included a total of 54 articles in the systematic review and 33 articles, containing 55 models, in the meta-analysis. Due to variations in study design and the measurement of both PA and SOSU, reaching a clear conclusion about how different types of functional SOSU relate to PA remains challenging. Overall, however, our findings suggest a positive relationship between functional SOSU and PA.

Almost half of all studies (*n* = 25) in the systematic review investigated SSPA and PA. While most studies found a significant positive association, two studies [36, 55] reported a significant negative association, and one study found both positive and negative associations [56]. Zhou et al. found that family SSPA was positively associated with PA, whereas friend SSPA was negatively associated [56]. These findings offer moderate evidence that higher levels of SSPA, whether from multiple sources or specifically from family, are linked to increased PA. The link between SOSU from friends and PA was less consistent; however, two longitudinal studies identified a significant positive association, indicating a relationship that appears to have been stable across time [25, 46]. The meta-analysis provided additional statistical insight into the inconsistencies regarding the sources of SSPA and its association with PA, revealing that whether support came from family or friends did not significantly influence the strength of the relationship. Furthermore, the meta-analysis allowed us to further quantify the evidence concerning SSPA and PA: based on 28 models, we found a small but significant positive effect (β = 0.12, 95% CI: 0.06–0.17) of the former on the latter. This association remained stable when individual studies were removed from the analysis, although substantial heterogeneity was observed between studies. Some publication bias was found, however, suggesting that the true effect size may be slightly smaller than reported.

A stronger association between SOSU and PA emerged when we focused specifically on general SOSU. In the systematic review, the evidence here was notably consistent, with nearly all studies reporting significant associations and only one failing to do so [52]. However, two studies reported significant negative associations [48, 52]. One of these further distinguished between receiving and providing support, finding that receiving support was linked to lower PA levels while providing support was associated with higher PA levels [49], suggesting that those in need of support may be less physically capable, whereas those able to give support tend to be more active.

The forest plots also report a positive association between general SOSU and PA, but only the findings in which PA was measured continuously were significant. The meta-analysis revealed a significant positive association between general SOSU and PA, with a pooled standardised beta coefficient of 0.35 (95% CI: 0.16–0.53). This reflects a moderate effect size, suggesting that higher levels of general SOSU are reliably linked to increased PA.

Although the meta-regression indicated larger adjusted effect sizes for studies measuring general SOSU, this finding should not be interpreted as evidence that general SOSU is inherently more influential than SSPA. Instead, the discrepancy between the subgroup meta-analyses and the meta-regression probably reflects substantial methodological heterogeneity between studies, differences in design and measurement, and the broader conceptual scope of general SOSU. In this systematic review, the general SOSU category comprised studies assessing overall functional SOSU, as well as studies employing composite instruments such as the DSSI, which combine the functional and structural aspects of SOSU into a single score. Consequently, the general SOSU category captured a broader social resource than SSPA alone. This broader operationalisation may partly account for the larger adjusted effect sizes observed in the meta-regression, since structural aspects of SOSU – such as the number of people providing emotional SOSU – have also been linked to health behaviours, including PA. Therefore, the observed difference may reflect differences in construct operationalisation rather than general SOSU being inherently more influential than SSPA.

The significant associations found in studies using continuous PA outcomes, compared to the less consistent findings in cases involving binary measures, suggest that the way PA is operationalised can meaningfully influence results. This difference likely reflects more than just outcome type. Binary outcome studies were few and varied in the PA category addressed (e.g. LTPA vs. MVPA), measurement tools used and cutoff thresholds applied, which may explain the lack of significant associations. Moreover, binary classifications typically assume a simple linear relationship between variables, which might lead studies to overlook more complex or nonlinear patterns. In contrast, continuous measures allow more nuanced analysis and showed more consistent positive relationships. These findings highlight the importance of measurement choices and consistent operationalisation and analytical approaches in research on SOSU and PA.

A majority of the included studies on other forms of functional SOSU indicated a positive relationship: emotional support largely showed a significant positive association with PA [34, 37, 39, 60, 66–69], while support in the form of companionship (e.g. doing activities together) had a particularly strong positive relationship with PA. Five out of six studies on companionship SOSU found significant positive effects, indicating that social interaction through PA is a key factor in its promotion, especially in community-dwelling older adults [28, 40, 73–75]. In contrast, the evidence for the effect of instrumental SOSU was mixed: of the seven studies identified, four reported a significant positive association [34, 37, 39, 72], while two reported a significant negative association [70, 71] and one found no significant results [32]. This inconsistency may be due to the different types of instrumental support provided (e.g. financial support). Only two studies focused on informational SOSU, with one finding a significant positive association [60] and the other reporting no significant association [70]. Notably, no studies examined the effect of validation support.

With regard to specific PA categories, findings from both the systematic review and the meta-analysis indicate a positive association between SOSU and LPA and SOSU and LTPA. In particular, LPA – especially walking – appears to be positively associated with functional SOSU [29, 41–43, 56, 61, 72, 74], a trend further reinforced by the results of the meta-analysis. Both LTPA and LPA consistently show positive associations with SOSU. It is important to note that LTPA often includes walking for leisure, which overlaps with LPA, highlighting the interconnected nature of these activity domains. In contrast, the evidence regarding MVPA remained inconclusive across both the systematic review and the meta-analysis. Although forest plots indicated a small to moderate positive association between SOSU and continuous MVPA outcomes, no significant relationship emerged for binary MVPA outcomes. The discrepancy between significant findings in studies using continuous PA measures and non-significant findings in those using binary measures further highlights how the definition and measurement of PA can substantially influence study results. Evidence of gender-related differences in the association between SOSU and PA was found in the systematic review, with gender-stratified results generally showing stronger associations in women [30, 31, 33, 34, 47]. This is consistent with previous research indicating that SOSU is more strongly and consistently associated with self-rated health and health behaviours in women than in men [80, 81]. One possible explanation is that women may be more likely to engage in socially oriented or group-based physical activities [82], in which SOSU is more directly embedded and therefore more influential. However, given the small number of studies reporting gender-stratified analyses and the inability to conduct formal subgroup analyses, these findings should be interpreted with caution.

Beyond individual characteristics, the broader social context in which older adults are embedded has also changed substantially over recent decades, which may have influenced the relationship between SOSU and PA. Many countries have expanded community services for older adults, organised PA programmes, and broader healthy ageing initiatives. At the same time, demographic changes, such as smaller family networks and greater geographical dispersion of relatives, may have altered how SOSU influences health behaviours. While the included studies span the period from 1993 to 2025, more than two-thirds (68.5%) were published after 2015, indicating that the evidence base primarily reflects a period in which formal support systems were already relatively well established in many of the included countries. Nevertheless, future research should investigate whether broader societal and welfare-state contexts moderate the association between SOSU and PA.

Longitudinal research highlights that even when PA is adopted later in life, it can still yield considerable health benefits for older adults [7]. This is echoed by the findings of a recent cohort study [83], which showed that engaging in any form of LTPA was associated with reduced mortality, regardless of intensity. Although more vigorous activities were linked to even greater reductions in mortality risk, the study demonstrated that all types of LTPA, including walking, were beneficial. These results emphasise the importance of promoting enjoyable forms of PA that older adults are likely to maintain. The centrality of enjoyment and sustainability is further supported by a recent scoping review of 25 qualitative studies on PA in adults aged 70 and over [84], which underscored the value of the ‘social opportunity’ offered by PA. Exercising with others increased participants’ enjoyment and reduced loneliness, benefits often valued more than the activity itself. These findings are in line with those of Steinhoff and Reiner, who identified a possible bidirectional relationship between PA and SOSU [9]. Additionally, a 2011 systematic review found that PA interventions were more effective when they incorporated elements of SOSU, further emphasising the need to consider social dimensions in promoting PA among older adults [85].

These findings provide a strong basis for the development of targeted public health interventions. Since even small increases in PA can benefit health and well-being, such interventions should prioritise accessible, low-impact PA programmes. These programmes can then serve as an entry point – helping older adults build confidence and capability – which may, over time, lead to their increased engagement in MVPA.

### Strengths and limitations

The present study is subject to some limitations that must be acknowledged. Firstly, this review intentionally excluded studies focusing on specific patient populations or individuals in institutional settings, thereby narrowing the scope of research included. As a result, the findings reflect only community-dwelling adults who were not part of defined clinical groups. This decision was deliberate, however, as the excluded populations often have distinct support needs and PA capacities that differ from the general older adult population. Secondly, the review was limited to articles published in English, potentially resulting in an under-representation of findings from non-English-speaking countries. This limitation is also reflected in the geographic distribution of the included studies, with only four studies originating from South America and none from Africa. Consequently, low- and middle-income countries may be underrepresented, limiting the generalisability of the findings across diverse cultural and socioeconomic contexts. This restriction to English-language publications, however, was necessary to minimise the risk of misinterpretation or bias due to the authors’ varying levels of proficiency in other languages. Previous research has shown, moreover, that excluding non-English articles generally has little impact on the overall results and conclusions of reviews [86, 87].

Thirdly, there was substantial heterogeneity across the included studies, particularly in how PA and SOSU were measured. This variability limited our ability to make direct comparisons and necessitated a primarily narrative and descriptive synthesis.

Regarding PA, studies employed subjective measures, objective measures (accelerometers), or combinations of both, introducing an additional source of methodological heterogeneity. Self-report instruments, such as CHAMPS and PASE, have been developed and validated for older adults and are practical to administer in large population-based studies, but remain susceptible to recall and social desirability bias [76]. Accelerometer-based measures avoid some of these biases but are sensitive to methodological decisions, including wear-time criteria, epoch length, valid-day definitions, and intensity cut-points, all of which may influence estimates of PA [88]. Among the 54 included studies, only seven incorporated accelerometer-based assessments. Of these, four relied exclusively on accelerometer-derived outcomes, whereas the remaining three combined objective measurements with self-reported PA data. We did not extract this level of protocol detail from the accelerometer-based studies, and were therefore unable to assess directly how much such differences might have contributed to the heterogeneity of findings in the systematic review. To holistically capture PA, objective measures were included in the systematic review, however, the meta-analysis was restricted to studies using subjective measures of PA to ensure statistical comparability.

Furthermore, with only five of the included studies using a longitudinal design, it was not possible to assess the causality of the relationship between SOSU and PA. The high heterogeneity across studies also limited our meta-analysis to the assessment of the relationships between general SOSU and subjective PA and SSPA and subjective PA; such heterogeneity is nonetheless common in meta-analyses of self-reported behavioural and observational data due to variations in populations, contexts and measurement methods [78]. Ultimately, just 55 models from 33 studies met the criteria for inclusion in the meta-regression based on their similarity in measurements. More detailed subgroup analyses and the analysis of dissimilar measurements were not feasible, so these findings remain context-free and pertain to a narrow selection of measures. Gender differences could be meaningfully assessed through subgroup analysis in future research. Lastly, the value of this study is limited by publication bias. Although statistical techniques were used to detect and adjust for this bias, it remains possible that studies reporting stronger associations between SOSU and PA were more likely to be published, potentially leading to an overestimation of the true effects.

Despite its limitations, this study has notable strengths. The narrowly defined research question and strict inclusion and exclusion criteria have allowed us to provide a systematic overview of the relationship between functional SOSU and PA in community-dwelling older adults. One major strength is the exclusive focus on functional, rather than structural, SOSU, which allows clearer identification of which qualitative aspects of support promote or potentially hinder PA. This distinction is valuable for guiding future research and informing interventions, particularly by highlighting the role of SOSU quality in promoting PA. Although structural elements of SOSU are also associated with PA, these operate through different mechanisms, so it is important to differentiate between structural and functional dimensions. Hence, this review offers conceptual clarity by clearly distinguishing between structural and functional aspects, the latter of which are often overlooked in the literature. Furthermore, by not only providing a narrative systematic review but also conducting a meta-analysis on the association between general SOSU and SSPA, on the one hand, and subjective PA, on the other, we were able to further quantify the results regarding these support types. This combination of narrative and quantitative synthesis strengthens the robustness of our conclusions and provides a more comprehensive picture of the evidence.

## Implications for future research

Firstly, future research should focus on using consistent, validated tools to measure both SOSU and PA, which would improve comparability across studies. To better understand how different types of SOSU relate to various PA categories, researchers should employ more detailed and precise instruments to assess PA, such as accelerometers or comprehensive self-report scales. This is crucial given the overlapping characteristics of LTPA and LPA, as well as the mixed evidence regarding MVPA.

Secondly, continuous outcome measures for PA should be prioritised in future research, as they provide more sensitivity than binary measures and allow for detailed statistical analysis. This is particularly important in studying older adults, whose activity levels often vary gradually rather than falling neatly into ‘active’ or ‘inactive’ categories. Continuous measures better capture this variation, making it easier to detect meaningful associations with SOSU and ensuring more accurate, age-relevant insights. In contrast, the binary measures employed in the analysed studies captured outcomes based on the threshold of *minimal* recommended PA (i.e. engaging in activity for 15 min three times per week). These thresholds might be achieved without reliance on SOSU and, at least in part, through the daily routines of older adults, while SOSU may be more effective in motivating older adults to exercise above and beyond such a minimum. Binary measures building on public recommendations may be useful for informing public health policies, but they do not discriminate between activity levels considered achievable for most healthy people and intense activity as engaged in by some individuals. Researchers will thus have to weigh the benefits and downsides of the different measures depending on their study aims.

Thirdly, future studies should ensure the use of measurement tools that reflect the lived experiences and physical capacities of older adults. For instance, the SSES [54] primarily captures exercise-related PA and may overlook important areas such as household- or transport-related activities, which older adults may not perceive as ‘exercise’. A 2017 review already emphasised the importance of adapting and validating tools such as the SSES for use in this demographic [8]; wider adoption of such age-specific instruments would be valuable, given the particular physical abilities and activity patterns of older adults. In addition, there is an urgent need to develop and validate SOSU measures that specifically address the contexts and experiences of older people, who often draw on different sources of support and have different support needs from younger populations [8, 9]. Furthermore, future research, particularly in older adult populations, should distinguish explicitly between received and provided SOSU. Rather than combining them into composite scores, these dimensions should be reported separately to allow clearer interpretation of their distinct effects.

Fourthly, no studies examined validation support. Therefore, future research should address this understudied dimension of functional SOSU. As validation support operates through mechanisms such as social comparison and feedback, it may be a particularly relevant pathway for promoting PA, and therefore warrants greater attention in both observational and intervention research.

Lastly, additional longitudinal research is essential to determine whether a causal relationship exists between SOSU and PA. Only five studies included in this review used a longitudinal design, limiting our ability to assess the directionality of the associations found. Gaining insight into how various forms of SOSU influence PA over time in older adults would help guide the development of more targeted and effective interventions.

## Conclusion

In this study, we found a small but statistically significant positive relationship between SSPA and PA and a medium-to-strong significant positive relationship between general SOSU and PA. Furthermore, in the systematic review of 54 articles, we mostly found a positive association between functional aspects of SOSU and PA. These findings suggest that functional SOSU is an important facilitator of PA in older adults. However, there is a clear need for better-designed studies that use more standardised and age-appropriate measures of both SOSU and PA in order to reduce heterogeneity across studies. In addition, more longitudinal research is required to adequately assess the relationship over time and strengthen causal implications. Existing research has found, that engaging in PA can enhance social integration in older adults [9, 84], thus suggesting that the relationship may be bidirectional in nature. Public health interventions could benefit from this mutuality: by increasing PA levels in older adults through group programmes, public health authorities could also enhance the SOSU available to this demographic, which, in turn, stands not only to improve PA adherence but also to promote better physical and mental health overall. Thus, two health challenges can be addressed through a single approach. Promoting PA in older adults may support not only physical health through increased activity levels, but also broader healthy ageing outcomes such as social participation and well-being.

## Supplementary Information


Supplementary Material 1.



Supplementary Material 2.


## Data Availability

The datasets used and/or analysed during the current study are available from the corresponding author on reasonable request. All data generated or analysed during this study are included in this published article.

## Abbreviations

PA: Physical Activity
LTPA: Leisure time physical activity
MVPA: Moderate to vigorous physical activity
LPA: Light physical activity
TPA: Total physical activity
SOSU: Social support
SSPA: Social support for physical activity

## References

[1] United Nations Department of Economic and Social Affairs. Population Division. World Population Prospects 2022. 2022.

[2] World Health Organization. Global status report on physical activity 2022. 2022. Available from: https://www.who.int/publications-detail-redirect/9789240059153. [Cited 1 Dec 2022].

[3] Cunningham C, O’ Sullivan R, Caserotti P, Tully MA. Consequences of physical inactivity in older adults: A systematic review of reviews and meta-analyses. Scand J Med Sci Sports. 2020;30(5):816–27. 10.1111/sms.13616. 32020713

[4] Lee IM, Shiroma EJ, Lobelo F, Puska P, Blair SN, Katzmarzyk PT. Effect of physical inactivity on major non-communicable diseases worldwide: an analysis of burden of disease and life expectancy. Lancet. 2012;380(9838):219–29. 10.1016/S0140-6736(12)61031-9. 22818936 PMC3645500

[5] Ding D, Lawson KD, Kolbe-Alexander TL, Finkelstein EA, Katzmarzyk PT, van Mechelen W, et al. The economic burden of physical inactivity: a global analysis of major non-communicable diseases. Lancet. 2016;388(10051):1311–24. 10.1016/S0140-6736(16)30383-X. 27475266

[6] World Health Organization. WHO guidelines on physical activity and sedentary behaviour. Copenhagen: World Health Organization. Regional Office for Europe. 2020. Available from: https://apps.who.int/iris/handle/10665/353806. [Cited 1 Dec 2022].

[7] Hamer M, Lavoie KL, Bacon SL. Taking up physical activity in later life and healthy ageing: the English longitudinal study of ageing. Br J Sports Med. 2014;48(3):239–43. 10.1136/bjsports-2013-092993 . PubMed PMID: 24276781. 24276781 PMC3913225

[8] Lindsay Smith G, Banting L, Eime R, O’Sullivan G, van Uffelen JGZ. The association between social support and physical activity in older adults: a systematic review. Int J Behav Nutr Phys Act. 2017;14(1):56. 10.1186/s12966-017-0509-8. 28449673 PMC5408452

[9] Steinhoff P, Reiner A. Physical activity and functional social support in community-dwelling older adults: a scoping review. BMC Public Health. 2024;24(1):1355. 10.1186/s12889-024-18863-6. 38769563 PMC11103817

[10] Cohen S, Wills TA. Stress, social support, and the buffering hypothesis. Psychol Bull. 1985;98(2):310–57. 10.1037/0033-2909.98.2.310. 3901065

[11] Wills TA, Shinar O. Measuring perceived and received social support. Social support measurement and intervention: A guide for health and social scientists. New York, NY, US: Oxford University Press; 2000. pp. 86–135. 10.1093/med:psych/9780195126709.003.0004.

[12] Golaszewski NM, Bartholomew JB. The Development of the Physical Activity and Social Support Scale. J Sport Exerc Psychol. 2019;41(4):215–29. 10.1123/jsep.2018-0234. 31461243

[13] Deeks JJ, Higgins JP, Altman DG, on behalf of the Cochrane Statistical Methods Group. Analysing data and undertaking meta-analyses. In: Higgins JPT, Thomas J, Chandler J, Cumpston M, Li T, Page MJ, editors. Cochrane Handbook for Systematic Reviews of Interventions. 1st edn. Wiley; 2019. pp. 241–84. Available from: https://onlinelibrary.wiley.com/. [Cited 5 Feb 2025].

[14] Page MJ, McKenzie JE, Bossuyt PM, Boutron I, Hoffmann TC, Mulrow CD, et al. The PRISMA 2020 statement: an updated guideline for reporting systematic reviews. BMJ. 2021;372:n71. 10.1136/bmj.n71 . PubMed PMID: 33782057. 33782057 PMC8005924

[15] World Health Organization. Ageing and health. 2025. Ageing and health. Available from: https://www.who.int/news-room/fact-sheets/detail/ageing-and-health. [Cited 9 Jul 2026].

[16] Laird Y, Fawkner S, Kelly P, McNamee L, Niven A. The role of social support on physical activity behaviour in adolescent girls: a systematic review and meta-analysis. Int J Behav Nutr Phys Act. 2016;13(1):79. 10.1186/s12966-016-0405-7. 27387328 PMC4937604

[17] Ouzzani M, Hammady H, Fedorowicz Z, Elmagarmid A. Rayyan—a web and mobile app for systematic reviews. Syst Rev. 2016;5(1):210. 10.1186/s13643-016-0384-4. 27919275 PMC5139140

[18] Wells G, Shea B, O’Connell D, Peterson J, Welch V, Losos M et al. The Newcastle-Ottawa Scale (NOS) for assessing the quality of nonrandomised studies in meta-analyses. 2014. Available from: https://www.ohri.ca/programs/clinical_epidemiology/oxford.asp. [Cited 22 Aug 2024].

[19] Mohd TAMT, Yunus RM, Hairi F, Hairi NN, Choo WY. Social support and depression among community dwelling older adults in Asia: a systematic review. BMJ Open. 2019;9(7):e026667. 10.1136/bmjopen-2018-026667 . PubMed PMID: 31320348. PMC6661578 31320348

[20] Reiner A, Steinhoff P. The association of social networks and depression in community-dwelling older adults: a systematic review. Syst Rev. 2024;13(1):161. 10.1186/s13643-024-02581-6. 38902787 PMC11188217

[21] Shamsrizi P, Gladstone BP, Carrara E, Luise D, Cona A, Bovo C, et al. Variation of effect estimates in the analysis of mortality and length of hospital stay in patients with infections caused by bacteria-producing extended-spectrum beta-lactamases: a systematic review and meta-analysis. BMJ Open. 2020;10(1):e030266. 10.1136/bmjopen-2019-030266 . PubMed PMID: 31964661. 31964661 PMC7044956

[22] Wagner P, Duan YP, Zhang R, Wulff H, Brehm W. Association of psychosocial and perceived environmental factors with park-based physical activity among elderly in two cities in China and Germany. BMC Public Health. 2020;20(1):55. 10.1186/s12889-019-8140-z. PMC6961356 31937268

[23] Bopp M, Wilcox S, Oberrecht L, Kammermann S, McElmurray CT. Correlates of strength training in older rural African American and Caucasian women. Women Health. 2004;40(1):1. 10.1300/J013v40n01_01. Located at: rayyan-881799550. 15778129

[24] Cousins SO. Exercise cognition among elderly women. J Appl Sport Psychol. 1996;8(2):131–45. 10.1080/10413209608406472.

[25] Harvey IS, Alexander K. Perceived Social Support and Preventive Health Behavioral Outcomes among Older Women. J Cross Cult Gerontol. 2012;27(3):3. 10.1007/s10823-012-9172-3. PMC3424611 22836374

[26] Marthammuthu T, Hairi FM, Choo WY, Salleh NAM, Hairi NN. The Prevalence and Association Between Social Support and Physical Activity Among the Rural Community-Dwelling Older Women in a Southeast Asian Country. J Aging Phys Act. 2023;31(4):611–20. 10.1123/japa.2022-0201. 36649719

[27] Wilcox S, Bopp M, Oberrecht L, Kammermann SK, McElmurray CT. Psychosocial and perceived environmental correlates of physical activity in rural and older African American and white women. Journals Gerontol Ser B: Psychol Sci Social Sci. 2003;58(6):P329–37. 10.1093/geronb/58.6.P329. Located at: rayyan-881799423. 14614117

[28] Salvador EP, Florindo AA, Reis RS, Costa EF. Perception of the environment and leisure-time physical activity in the elderly. Rev Saude Publica. 2009;43:972-80. 10.1590/S0034-89102009005000082. 20027502

[29] Abdi A, O’Hern S. Understanding leisure walking behaviour among recently retired older adults in Tehran: Gender-specific influences and regional implications. J Transp Health. 2025;42:102032. 10.1016/j.jth.2025.102032.

[30] Blakoe M, Petrova D, Garcia-Retamero R, Gonçalves K, Catena A, Ramírez Hernández JA, et al. Sex Moderates the Relationship Between Social Support and Cardiovascular Prevention Behaviors in Middle-aged and Older Adults. Ann Behav Med. 2023;57(10):877–87. 10.1093/abm/kaad030. 37357373

[31] Kaplan MS, Newsom JT, McFarland BH, Lu L. Demographic and psychosocial correlates of physical activity in late life. Am J Prev Med. 2001;21(4):4. 10.1016/S0749-3797(01)00364-6. 11701302

[32] Komazawa Y, Murayama H, Harata N, Takami K. Giancarlos Troncoso Parady. Role of Social Support in the Relationship Between Financial Strain and Frequency of Exercise Among Older Japanese: A 19-year Longitudinal Study. J Epidemiol. 2021;31(4):265–71. 10.2188/jea.JE20190248. Located at: rayyan-881799377. 32307351 PMC7940977

[33] Lian WM, Gan GL, Pin CH, Wee S, Ye HC. Correlates of leisure-time physical activity in an elderly population in Singapore. Am J Public Health. 1999;89(10):1578–80. 10.2105/AJPH.89.10.1578. 10511845 PMC1508788

[34] Loprinzi PD, Joyner C. Source and size of emotional and financial-related social support network on physical activity behavior among older adults. J Phys Activity Health. 2016;13(7):776–9. 10.1123/jpah.2015-0629. Located at: rayyan-881799411. 26900842

[35] Oka K, Shibata A. Determinants of meeting the public health recommendations for physical activity among community-dwelling elderly Japanese. Curr Aging Sci. 2012;5(1):58–65. Located at: rayyan-881799385. 21762088 10.2174/1874609811205010058

[36] van Luchene P, Detemmerman F, Delens C. The Influence of COVID-19 Lockdown on Physical Activity Sedentary Behavior and Social Support Specific to Physical Activity Among Belgian Adults. Front SPORTS Act LIVING. 2021;3:716386. Located at: rayyan-881799417. 34617010 10.3389/fspor.2021.716386 PMC8488097

[37] Yamakita M, Kanamori S, Kondo N, Kondo K. Correlates of Regular Participation in Sports Groups among Japanese Older Adults: JAGES Cross–Sectional Study. PLoS ONE. 2015;10(10):10. 10.1371/journal.pone.0141638. PMC4626107 26512895

[38] Gothe NP. Correlates of Physical Activity in Urban African American Adults and Older Adults: Testing the Social Cognitive Theory. Ann Behav Med. 2018;52(9):9. 10.1093/abm/kax038. 30124762 PMC6128373

[39] Zambrano Garza E, Pauly T, Choi Y, Murphy RA, Linden W, Ashe MC, et al. Everyday Social Support for Health Behaviours in Older Adults during Times of Challenge: Evidence from Daily Life Assessments. Can J Aging / La Revue canadienne du vieillissement. 2024;44(2):215–24. 10.1017/S0714980824000412. 39641444

[40] Zambrano Garza E, Pauly T, Choi Y, Murphy RA, Linden W, Ashe MC, et al. Social resources for everyday physical activity during times of chronic stress. Couple Family Psychology: Res Pract. 2025;14(4):328–40. 10.1037/cfp0000277.

[41] Carlson JA, Sallis JF, Conway TL, Saelens BE, Frank LD, Kerr J et al. Interactions between psychosocial and built environment factors in explaining older adults’ physical activity. Preventive Medicine. 2012;Special Section: Complementary and Alternative Medicine II54(1):68–73. 10.1016/j.ypmed.2011.10.004. PMC3254837 22027402

[42] Thornton CM, Kerr J, Conway TL, Saelens BE, Sallis JF, Ahn DK, et al. Physical Activity in Older Adults: an Ecological Approach. Ann Behav Med. 2017;51(2):2. 10.1007/s12160-016-9837-1. PMC5373946 27680568

[43] Chen S, Calderón-Larrañaga A, Saadeh M, Dohrn IM, Welmer AK. Correlations of Subjective and Social Well-Being With Sedentary Behavior and Physical Activity in Older Adults—A Population-Based Study. Journals Gerontology: Ser A. 2021;76(10):1789–95. 10.1093/gerona/glab065. PMC8436992 33674835

[44] Manz K, Mensink GB, Jordan S, Schienkiewitz A, Krug S, Finger JD. Predictors of physical activity among older adults in Germany: a nationwide cohort study. BMJ Open. 2018;8(5):e021940. 10.1136/bmjopen-2018-021940. Located at: rayyan-881799477. 29743332 PMC5942462

[45] Smith GSE, Moyle W, Burton NW. The Relationship between Social Support for Physical Activity and Physical Activity across Nine Years in Adults Aged 60–65 Years at Baseline. Int J Environ Res Public Health. 2023;20(5):5. 10.3390/ijerph20054531. PMC10002128 36901538

[46] Warner LM, Ziegelmann JP, Schüz B, Wurm S, Schwarzer R. Synergistic Effect of Social Support and Self-Efficacy on Physical Exercise in Older Adults. J Aging Phys Act. 2011;19(3):249–61. 10.1123/japa.19.3.249. 21727305

[47] Chan YY, Lim KK, Omar MA, Mohd Yusoff MF, Sooryanarayana R, Ahmad NA, et al. Prevalence and factors associated with physical inactivity among older adults in Malaysia: A cross-sectional study. Geriatr Gerontol Int. 2020;20(S2):49–56. 10.1111/ggi.13977. 33370865

[48] Gomes M, Figueiredo D, Teixeira L, Poveda V, Paúl C, Santos-Silva A, et al. Physical inactivity among older adults across Europe based on the SHARE database. Age Ageing. 2017;46(1):71–7. 10.1093/ageing/afw165. 28181637 PMC6402309

[49] Hwang S, Yi ES, Breaking Barriers B, Habits. Psychological Analysis of the Relationship Between Perceived Barriers, Financial Burden, and Social Support on Exercise Adherence Among Adults Aged 50 and Older in South Korea. Healthcare. 2025;13(12):1469. 10.3390/healthcare13121469. 40565495 PMC12193192

[50] Jin Y, Yu R, Si H, Bian Y, Qiao X, Ji L, et al. Effects of social support on frailty trajectory classes among community-dwelling older adults: The mediating role of depressive symptoms and physical activity. Geriatr Nurs. 2022;45:39–46. 10.1016/j.gerinurse.2022.02.029. 35303526

[51] Kang HW, Park M, Wallace (Hernandez) JP. The impact of perceived social support, loneliness, and physical activity on quality of life in South Korean older adults. J Sport Health Sci. 2018;7(2):2. 10.1016/j.jshs.2016.05.003. PMC6180534 30356448

[52] Lieber SB, Moxley J, Mandl LA, Reid MC, Czaja SJ. Social support and physical activity: does general health matter? Eur Rev Aging Phys Act. 2024;21(1):16. 10.1186/s11556-024-00347-6. 38902616 PMC11188280

[53] Yi X, Pope Z, Gao Z, Wang S, Pan F, Yan J, et al. Associations between individual and environmental factors and habitual physical activity among older Chinese adults: A social–ecological perspective. J Sport Health Sci. 2016;5(3):315–21. 10.1016/j.jshs.2016.06.010. 30356490 PMC6188579

[54] Sallis JF, Grossman RM, Pinski RB, Patterson TL, Nader PR. The development of scales to measure social support for diet and exercise behaviors. Prev Med. 1987;16(6):825–36. 10.1016/0091-7435(87)90022-3. 3432232

[55] Park CH, Elavsky S, Koo KM. Factors influencing physical activity in older adults. J Exerc Rehabil. 2014;10(1):1. 10. 12965/jer.140089 PubMed PMID: 24678504; PubMed Central PMCID: PMC3952835. 24678504 10.12965/jer.140089 PMC3952835

[56] Zhou J, Yang C, Yu J, Zhao X, Wu J, Liu Z, et al. The Influence of Social Support on Leisure-Time Physical Activity of the Elderly in the Chinese Village of Fuwen. Healthcare. 2023;11(15):2193. 10.3390/healthcare11152193. 37570433 PMC10418849

[57] Bakhtari F, Ahmad B, Aminisani N, Gilani N, Allahverdipou H. Psychological, social, and environmental predictors of physical activity among older adults: The socio-ecological approach using structural equation modeling analysis. BJHPA. 2019;11(2):2. 10.29359/BJHPA.11.2.12.

[58] Lee YH, Fan SY. Psychosocial and environmental factors related to physical activity in middle-aged and older adults. Sci Rep. 2023;13(1):1. 10.1038/s41598-023-35044-4. 37179430 PMC10182976

[59] Sirotiak Z, Brellenthin AG, Hariharan A, Welch AS, Meyer JD, Franke WD. Psychological correlates of physical activity among adults living in rural and urban settings. Front Psychol. 2024;15. 10.3389/fpsyg.2024.1389078. PMC11039787 38659683

[60] Newsom JT, Shaw BA, August KJ, Strath SJ. Physical activity–related social control and social support in older adults: Cognitive and emotional pathways to physical activity. J Health Psychol. 2018;23(11):11. 10.1177/1359105316656768. 27469008

[61] Corseuil Giehl MW, Hallal PC, Brownson RC, d’Orsi E. Exploring Associations Between Perceived Measures of the Environment and Walking Among Brazilian Older Adults. J Aging Health. 2017;29(1):45–67. 10.1177/0898264315624904. 26754197

[62] de Sousa BA, da Silva FC, Ribeiro ÍL, de Lima DB, da Silva R, Sociodemographic. Anthropometric, Functional and Psychosocial Factors Associated with Physical Activity in Older Adults. Ageing Int. 2021 Aug;2. 10.1007/s12126-021-09450-w.

[63] Safavi SR, Sadeghi R, Jamshidi E, Tedadi Y, Yaseri M, Shati M, et al. The association between self-efficacy and social support with physical activity in older adults: a cross-sectional study. Sci Rep. 2025 Oct 21;15(1):36769. 10.1038/s41598-025-20717-z. PMC12540690 41120545

[64] Steijvers LC, Wagner S, Bruinsma J, Hoebe CJ, Dukers-Muijrers NH. Social networks and levels of physical activity in Dutch adults – the SaNAE study. International Journal of Sport and Exercise Psychology. 2025 Oct 21;0(0):1–21. 10.1080/1612197X.2025.2570184.

[65] Kim Y, Kosma M. Psychosocial and Environmental Correlates of Physical Activity Among Korean Older Adults. Res Aging. 2013;35(6):6. 10.1177/0164027512462412.

[66] Krause N, Goldenhar L, Liang J, Jay G, Maeda D. Stress and exercise among the Japanese elderly. Soc Sci Med. 1993;36(11):1429–41. 10.1016/0277-9536(93)90385-H. 8511631

[67] Lee HH, Kim ES, Kim Y, Conroy DE, VanderWeele TJ. Exploring novel determinants of exercise behavior: a lagged exposure-wide approach. Ann Behav Med. 2025;59(1):kaae082. 10.1093/abm/kaae082. 39756405 PMC11700616

[68] Watt RG, Heilmann A, Sabbah W, Newton T, Chandola T, Aida J, et al. Social relationships and health related behaviors among older US adults. BMC Public Health. 2014;14(1):533. 10.1186/1471-2458-14-533. 24885507 PMC4046043

[69] Yuan S, Elam KK, Johnston JD, Lin HC, Chow A. The Relationship Between Three Sources of Social Support and Physical Activity Level in Middle-Aged and Older Adults. Int J Aging Hum Dev. 2025;101(2):210–24. 10.1177/00914150241267994. 39105263

[70] Zimmer C, McDonough MH. Social Support and Physical Activity in Older Adults: Identifying Predictors Using Data From the Canadian Longitudinal Study on Aging. J Aging Phys Act. 2021;30(1):136–47. 10.1123/japa.2020-0393. 34348225

[71] Perrino T, Brown SC, Huang S, Brown CH, Pérez Gómez G, Pantin H, et al. Depressive symptoms, social support, and walking among Hispanic older adults. J Aging Health. 2011;23(6):974-93. 10.1177/0898264311404235. PMC3812821 21508305

[72] Van Cauwenberg J, De Donder L, Clarys P, De Bourdeaudhuij I, Buffel T, De Witte N, et al. Relationships between the perceived neighborhood social environment and walking for transportation among older adults. Soc Sci Med. 2014;104:23–30. 10.1016/j.socscimed.2013.12.016. 24581058

[73] Böhm AW, Mielke GI, Cruz MF da, Ramires VV, Wehrmeister FC. Social Support and Leisure-Time Physical Activity Among the Elderly: A Population-Based Study. Journal of Physical Activity and Health. 2016 Jun 1;13(6):599–605. 10.1123/jpah.2015-0277. 26696310

[74] Ory MG, Towne SD, Won J, Forjuoh SN, Lee C. Social and environmental predictors of walking among older adults. BMC Geriatr. 2016;16(1):1. 10.1186/s12877-016-0327-x. 27553668 PMC4995768

[75] Shores KA, West ST, Theriault DS, Davison EA. Extra-Individual Correlates of Physical Activity Attainment in Rural Older Adults. J Rural Health. 2009;25(2):2. 10.1111/j.1748-0361.2009.00220.x. 19785589

[76] Kowalski K, Rhodes R, Naylor PJ, Tuokko H, MacDonald S. Direct and indirect measurement of physical activity in older adults: a systematic review of the literature. Int J Behav Nutr Phys Act. 2012;9(1):148. 10.1186/1479-5868-9-148. 23245612 PMC3549726

[77] Orsega-Smith EM, Payne LL, Mowen AJ, Ho CH, Godbey GC. The Role of Social Support and Self-Efficacy in Shaping the Leisure Time Physical Activity of Older Adults. J Leisure Res. 2007;39(4):4. 10.1080/00222216.2007.11950129.

[78] Migliavaca CB, Stein C, Colpani V, Barker TH, Ziegelmann PK, Munn Z, et al. Meta-analysis of prevalence: I2 statistic and how to deal with heterogeneity. Res Synthesis Methods. 2022;13(3):363–7. 10.1002/jrsm.1547. 35088937

[79] Afonso J, Ramirez-Campillo R, Clemente FM, Büttner FC, Andrade R. The Perils of Misinterpreting and Misusing Publication Bias in Meta-analyses: An Education Review on Funnel Plot-Based Methods. Sports Med. 2024;54(2):257–69. 10.1007/s40279-023-01927-9. 37684502 PMC10933152

[80] Caetano SC, Silva CM, Vettore MV. Gender differences in the association of perceived social support and social network with self-rated health status among older adults: a population-based study in Brazil. BMC Geriatr. 2013;13(1):122. 10.1186/1471-2318-13-122. 24229389 PMC4225700

[81] Denton M, Prus S, Walters V. Gender differences in health: a Canadian study of the psychosocial, structural and behavioural determinants of health. Soc Sci Med. 2004;58(12):2585–600. 10.1016/j.socscimed.2003.09.008. 15081207

[82] van Uffelen JGZ, Khan A, Burton NW. Gender differences in physical activity motivators and context preferences: a population-based study in people in their sixties. BMC Public Health. 2017;17(1):624. 10.1186/s12889-017-4540-0. 28676081 PMC5496591

[83] Watts EL, Matthews CE, Freeman JR, Gorzelitz JS, Hong HG, Liao LM, et al. Association of Leisure Time Physical Activity Types and Risks of All-Cause, Cardiovascular, and Cancer Mortality Among Older Adults. JAMA Netw Open. 2022;5(8):e2228510. 10.1001/jamanetworkopen.2022.28510. 36001316 PMC9403775

[84] Meredith SJ, Cox NJ, Ibrahim K, Higson J, McNiff J, Mitchell S, et al. Factors that influence older adults’ participation in physical activity: a systematic review of qualitative studies. Age Ageing. 2023;52(8):afad145. 10.1093/ageing/afad145. 37595070 PMC10438214

[85] Greaves CJ, Sheppard KE, Abraham C, Hardeman W, Roden M, Evans PH, et al. Systematic review of reviews of intervention components associated with increased effectiveness in dietary and physical activity interventions. BMC Public Health. 2011;11(1):119. 10.1186/1471-2458-11-119. 21333011 PMC3048531

[86] Nussbaumer-Streit B, Klerings I, Dobrescu AI, Persad E, Stevens A, Garritty C, et al. Excluding non-English publications from evidence-syntheses did not change conclusions: a meta-epidemiological study. J Clin Epidemiol. 2020;118:42–54. 10.1016/j.jclinepi.2019.10.011. 31698064

[87] Stridsberg SL, Richardson MX, Redekop K, Ehn M, Andersson SW. Gray Literature in Evaluating Effectiveness in Digital Health and Health and Welfare Technology: A Source Worth Considering. J Med Internet Res. 2022;24(3):e29307. 10.2196/29307. 35319479 PMC8987953

[88] Migueles JH, Cadenas-Sanchez C, Ekelund U, Delisle Nyström C, Mora-Gonzalez J, Löf M, et al. Accelerometer Data Collection and Processing Criteria to Assess Physical Activity and Other Outcomes: A Systematic Review and Practical Considerations. Sports Med. 2017;47(9):1821–45. 10.1007/s40279-017-0716-0. 28303543 PMC6231536

