# Supplementary material for: Functional social support and physical activity in community-dwelling older adults: a systematic review and meta-analysis
Source: BMC Public Health. 2026 Sep 1;26:2504. doi: 10.1186/s12889-026-28972-z (PMC13536801; doi:10.1186/s12889-026-28972-z)
Supplement: Supplementary file 1 [file 12889_2026_28972_MOESM2_ESM.pdf]

Table A1: PRISMA checklist

Section and Topic	Item #	Checklist item	Location where item is reported
**TITLE**			
Title	1	Identify the report as a systematic review.	Title
**ABSTRACT**			
Abstract	2	See the PRISMA 2020 for Abstracts checklist.	Abstract
**INTRODUCTION**			
Rationale	3	Describe the rationale for the review in the context of existing knowledge.	Introduction
Objectives	4	Provide an explicit statement of the objective(s) or question(s) the review addresses.	Introduction
**METHODS**			
Eligibility criteria	5	Specify the inclusion and exclusion criteria for the review and how studies were grouped for the syntheses.	Eligibility criteria (2.1)
Information sources	6	Specify all databases, registers, websites, organisations, reference lists and other sources searched or consulted to identify studies. Specify the date when each source was last searched or consulted.	Information sources and search strategy (2.2)
Search strategy	7	Present the full search strategies for all databases, registers and websites, including any filters and limits used.	Information sources and search strategy (2.2)
Selection process	8	Specify the methods used to decide whether a study met the inclusion criteria of the review, including how many reviewers screened each record and each report retrieved, whether they worked independently, and if applicable, details of automation tools used in the process.	Selection process (2.3)
Data collection process	9	Specify the methods used to collect data from reports, including how many reviewers collected data from each report, whether they worked independently, any processes for obtaining or confirming data from study investigators, and if applicable, details of automation tools used in the process.	Data collection process and data extraction (2.4)
Data items	10a	List and define all outcomes for which data were sought. Specify whether all results that were compatible with each outcome domain in each study were sought (e.g. for all measures, time points, analyses), and if not, the methods used to decide which results to collect.	Data collection process and data extraction (2.4), data items (2.5)
	10b	List and define all other variables for which data were sought (e.g. participant and intervention characteristics, funding sources). Describe any assumptions made about any missing or unclear information.	Data collection process and data extraction (2.4), data items (2.5)
Study risk of bias assessment	11	Specify the methods used to assess risk of bias in the included studies, including details of the tool(s) used, how many reviewers assessed each study and whether they worked independently, and if applicable, details of automation tools used in the process.	Risk of bias and quality assessment (2.6)
Effect measures	12	Specify for each outcome the effect measure(s) (e.g. risk ratio, mean difference) used in the synthesis or presentation of results.	Data extraction (2.4), Synthesis methods and analytical strategy (2.7)
Synthesis methods	13a	Describe the processes used to decide which studies were eligible for each synthesis (e.g. tabulating the study intervention characteristics and comparing against the planned groups for each synthesis (item #5)).	Synthesis methods and analytical strategy (2.7)

Section and Topic	Item #	Checklist item	Location where item is reported
	13b	Describe any methods required to prepare the data for presentation or synthesis, such as handling of missing summary statistics, or data conversions.	Synthesis methods and analytical strategy (2.7)
	13c	Describe any methods used to tabulate or visually display results of individual studies and syntheses.	Synthesis methods and analytical strategy (2.7)
	13d	Describe any methods used to synthesize results and provide a rationale for the choice(s). If meta-analysis was performed, describe the model(s), method(s) to identify the presence and extent of statistical heterogeneity, and software package(s) used.	Synthesis methods and analytical strategy (2.7)
	13e	Describe any methods used to explore possible causes of heterogeneity among study results (e.g. subgroup analysis, meta-regression).	Synthesis methods and analytical strategy (2.7)
	13f	Describe any sensitivity analyses conducted to assess robustness of the synthesized results.	Synthesis methods and analytical strategy (2.7)
Reporting bias assessment	14	Describe any methods used to assess risk of bias due to missing results in a synthesis (arising from reporting biases).	Synthesis methods and analytical strategy (2.7)
Certainty assessment	15	Describe any methods used to assess certainty (or confidence) in the body of evidence for an outcome.	na
**RESULTS**			
Study selection	16a	Describe the results of the search and selection process, from the number of records identified in the search to the number of studies included in the review, ideally using a flow diagram.	Figure 1
	16b	Cite studies that might appear to meet the inclusion criteria, but which were excluded, and explain why they were excluded.	Supplementary material
Study characteristics	17	Cite each included study and present its characteristics.	Results
Risk of bias in studies	18	Present assessments of risk of bias for each included study.	Supplementary material
Results of individual studies	19	For all outcomes, present, for each study: (a) summary statistics for each group (where appropriate) and (b) an effect estimate and its precision (e.g. confidence/credible interval), ideally using structured tables or plots.	Table 2 – 7
Results of syntheses	20a	For each synthesis, briefly summarise the characteristics and risk of bias among contributing studies.	Tables 2 – 11
	20b	Present results of all statistical syntheses conducted. If meta-analysis was done, present for each the summary estimate and its precision (e.g. confidence/credible interval) and measures of statistical heterogeneity. If comparing groups, describe the direction of the effect.	Figures 2 – 5, Tables 12 - 13
	20c	Present results of all investigations of possible causes of heterogeneity among study results.	Figure 6, Figure 7
	20d	Present results of all sensitivity analyses conducted to assess the robustness of the synthesized results.	Figures 2 – 5
Reporting biases	21	Present assessments of risk of bias due to missing results (arising from reporting biases) for each synthesis assessed.	Figure 6, Figure 7, Results: Asymmetry and publication bias

Section and Topic	Item #	Checklist item	Location where item is reported
Certainty of evidence	22	Present assessments of certainty (or confidence) in the body of evidence for each outcome assessed.	na
**DISCUSSION**			
Discussion	23a	Provide a general interpretation of the results in the context of other evidence.	Discussion
	23b	Discuss any limitations of the evidence included in the review.	Discussion, limitations
	23c	Discuss any limitations of the review processes used.	Discussion, limitations
	23d	Discuss implications of the results for practice, policy, and future research.	Limitations, implications for future research, conclusion
**OTHER INFORMATION**			
Registration and protocol	24a	Provide registration information for the review, including register name and registration number, or state that the review was not registered.	Methods
	24b	Indicate where the review protocol can be accessed, or state that a protocol was not prepared.	https://osf.io/uc5xe
	24c	Describe and explain any amendments to information provided at registration or in the protocol.	na
Support	25	Describe sources of financial or non-financial support for the review, and the role of the funders or sponsors in the review.	na
Competing interests	26	Declare any competing interests of review authors.	None of the authors have any interests to declare in relation to the review.
Availability of data, code and other materials	27	Report which of the following are publicly available and where they can be found: template data collection forms; data extracted from included studies; data used for all analyses; analytic code; any other materials used in the review.	Upon request

From: Page MJ, McKenzie JE, Bossuyt PM, Boutron I, Hoffmann TC, Mulrow CD, et al. The PRISMA 2020 statement: an updated guideline for reporting systematic reviews. BMJ 2021;372:n71. doi: 10.1136/bmj.n71. This work is licensed under CC BY 4.0. To view a copy of this license, visit https://creativecommons.org/licenses/by/4.0/

Table A2: Excluded studies and exclusion reasons

**Authors, year**	**Title**	**Exclusion reason**
Alley et al., 2025	The moderating effect of social support on the effectiveness of a web-based, computer-tailored physical activity intervention for older adults.	Ineligible study design
Asiamah et al., 2023	Associations between vigorous physical activity, social ties, social support, and self-reported health among older adults in Accra, Ghana.	No functional measure of SOSU
Awlime-Ableh et al., 2025	Determinants of physical activity levels and quality of life among older adults in Ghana: insights from cardiometabolic risk (CarMeR) study.	No functional measure of SOSU
Belgen Kaygısız et al., 2026	Physical Activity and Associated Barriers/Facilitators in Older Adults Living in Northern Cyprus.	No directionality assessed
Blakoe et al., 2023	Sex Moderates the Relationship Between Social Support and Cardiovascular Prevention Behaviors in Middle-aged and Older Adults.	Duplicate
Chan et al., 2020	Associations among living alone, social support and social activity in older adults	Ineligible analytical focus
Chen et al., 2015	Physical activity among older people living alone in Shanghai, China.	No functional measure of SOSU, no directionality assessed
Chia et al., 2023	Promoting Healthy Behaviors in Older Adults to Optimize Health-Promoting Lifestyle: An Intervention Study.	Ineligible study design
Cousins, 1995	Social support for exercise among elderly women in Canada.	Ineligible analytical focus
Denche-Zamorano et al., 2024	Influence of physical activity level and perceived social support on mental health and psychological distress in women with menopause problems	Age criteria not met
Dubash & Schafer, 2024	Social Network Negativity and Physical Activity: New Longitudinal Evidence for Young and Older Adults 2015-2018.	Age criteria not met
Eronen et al., 2012	Accumulation of disparity in physical activity in old age.	No functional measure of SOSU, ineligleble PA outcome
Gu et al., 2024	A serial mediation model of physical exercise and loneliness: the role of perceived social support and resilience.	No directionality assessed
Gyasi, 2019	Social support, physical activity and psychological distress among community-dwelling older ghanaians.	Ineligible analytical focus
Hall & McAuley, 2010	Individual, social environmental and physical environmental barriers to achieving 10 000 steps per day among older women.	No functional measure of SOSU
Harada et al., 2023	Social network and health behaviors among Japanese older adults: a three-wave longitudinal study.	No functional measure of SOSU

He & Wang, 2025	The influence of tai chi on the death anxiety of elderly people living alone: the chain mediating effect of social support and psychological capital	No directionality assessed
Hoehener et al., 2024	Investigating the Association Between Companionship and Behavior Concordance: A Dyadic Panel Study	No functional measure of SOSU
Kanamori et al., 2023	Factors Related to Physical Activity among Older Adults Who Relocated to a New Community after the Kumamoto Earthquake: A Study from the Viewpoint of Social Capital.	Duplicate
Kanamori et al., 2023	Factors related to physical activity among older adults who relocated to a New Community after the Kumamoto Earthquake: a study from the viewpoint of Social Capital.	No functional measure of SOSU
Kumar et al., 2022	Prevalence and determinants of social isolation among elderly in an urban slum of Raipur city—A community based crosssectional study.	No functional measure of SOSU
Kwag et al., 2011	The impact of perceived stress, Social Support, and Home-based physical activity on Mental Health among older adults.	Ineligible analytical focus
Lee & Fan, 2023	Psychosocial and environmental factors related to physical activity in middle-aged and older adults.	Duplicate
Lee & Oh, 2023	The relationship between social support and health-promoting lifestyle: Mediating role of health self-efficacy	Age criteria not met
Levy-Storms & Lubben, 2006	Network Composition and Health Behaviors among older Samoan women.	No functional measure of SOSU
Loprinzi & Crush, 2017	Source and Size of Social Support Network on Sedentary Behavior among older adults.	Ineligble PA outcome
Malek Rivan et al., 2022	Mediation effect of coping strategies on general psychological health among middle-aged and older adults during the COVID-19 pandemic.	Ineligible analytical focus
Manera et al., 2023	Social Isolation, Social Support and Loneliness and Their Relationship With Physical Activity and Physical Function Among Older People	No functional measure of SOSU
Marcelino et al., 2024	Frailty and social network among older Brazilian adults: evidence from ELSI-Brazil.	No directionality assessed
Marthammuthu et al., 2023	The Prevalence and Association Between Social Support and Physical Activity Among the Rural Community-Dwelling Older Women in a Southeast Asian Country.	Duplicate
Morris et al., 2010	Social determinants of physical exercise in older men in Jamaica.	Ineligible analytical focus
Mowen et al., 2007	The role of Park Proximity and Social Support in Shaping Park Visitation, Physical Activity, and Perceived Health among older adults.	Ineligible analytical focus

Mudrak et al., 2017	Social cognitive determinants of physical activity in Czech older adults.	No directionality assessed
Niedermeier et al., 2019	Is the effect of physical activity on quality of life in older adults mediated by Social Support?	Ineligible analytical focus
Oetzel et al., 2023	Enhancing health outcomes for Māori elders through an intergenerational cultural exchange and physical activity programme: a cross-sectional baseline study	No directionality assessed
Oh et al., 2020	Verification of the Mediating Effect of Social Support on physical activity and aging anxiety of Korean Pre-older adults.	Ineligible analytical focus
Okoye et al., 2022	Social Support, General Self-Efficacy, fear of falling, and physical activity among older adults in a Middle-Income Country.	Ineligible analytical focus
Oktaviani et al., 2022	Effects of Health-related behaviors and changes on successful aging among Indonesian older people.	Ineligible analytical focus
Pollak et al., 2024	Longitudinal Associations of Social Support and Gait Speed Decline in Aging.	Ineligble PA outcome
Pollak et al., 2024	Loneliness predicts decreased physical activity in widowed but not married or unmarried individuals.	No functional measure of SOSU
Potts et al., 1992	Social Support, Health-Promotive Beliefs, and Preventive Health behaviors among the Elderly.	Ineligible analytical focus
Purath et al., 2009	Physical fitness assessment of older adults in the primary care setting.	Ineligible analytical focus
Qu et al., 2023	Relationships among Square Dance, Group Cohesion, Perceived Social Support, and Psychological Capital in 2721 Middle-aged and older adults in China	Ineligible analytical focus
Qu et al., 2023	Relationships among Square Dance, Group Cohesion, Perceived Social Support, and Psychological Capital in 2721 Middle-Aged and Older Adults in China.	No directionality assessed
Reed et al., 2011	Social isolation and physical inactivity in older US adults: results from the Third National Health and Nutrition Examination Survey	No functional measure of SOSU
Riffle et al., 1989	Health-Promoting, Behaviors. Perceived Social Support, and self-reported Health of Appalachian Elderly	Ineligible analytical focus
Rowland et al., 2023	Depression, childhood trauma, and physical activity in older Indigenous Australians.	Duplicate
Ryu et al., 2023	Associations of physical activity and sedentary behavior with optimism and positive affect in Older Women.	Ineligible analytical focus
Ryu et al., 2023	Associations of Physical Activity and Sedentary Behavior with Optimism and Positive Affect in Older Women	Duplicate

Samal & Manchana, 2025	Interconnections of physical activity, cognition, functional health and social connectivity with perceived happiness among rural adults: Population based study	Age criteria not met
Seguin-Fowler et al., 2025	Association Between Physical Activity, Psychosocial Factors, and the Built Environment in Rural Adults.	Age criteria not met
Shen et al., 2025	The impact of internet use on physical activity among Chinese older adults: the mediating role of social support.	No functional measure of SOSU
Sjöberg et al., 2022	Factors associated with physical activity reduction in Swedish older adults during the first COVID-19 outbreak: a longitudinal population-based study.	No functional measure of SOSU
Smith et al., 2023	The Relationship between Social Support for Physical Activity and Physical Activity across Nine Years in Adults Aged 60-65 Years at Baseline.	Duplicate
Smith et al., 2023	Social Support for Physical Activity Over 9 Years in Adults Aged 60-65 Years at Baseline.	Duplicate
Smith et al., 2023	Frequency of Physical Activity Done with a Companion: Changes Over Seven Years in Adults Aged 60+ Living in an Australian Capital City.	No directionality assessed
Trujillo-Barberá et al., 2023	Predictors of physical activity in older adults 65 years and older: findings in health survey of the Valencian Community	No directionality assessed
Umstattd et al., 2006	Self-regulation for physical activity among older adults.	Ineligible analytical focus
Wang et al., 2022	Impact of Social Participation types on Depression in the Elderly in China: an analysis based on counterfactual causal inference.	Ineligible analytical focus
Wilcox et al. 2000	Determinants of leisure time physical activity in rural compared with urban older and ethnically diverse women in the United States.	Ineligble PA outcome
Witonsky et al., 2025	Joint Pain and Leisure-Time Physical Activity: Cross-Sectional Findings From the Cardiovascular Health Study.	No functional measure of SOSU
Wu et al., 2023	Mechanisms connecting square dance to sleep quality among middle-aged and older Chinese females: serial mediation roles of social support and depressive symptoms.	Age criteria not met
Yamagata et al., 2025	The Frailty, Fitness, and Psychophysical/Social Condition of Community-Dwelling Older Adults-Analysis of 5-Year Longitudinal Data.	No directionality assessed
Yuan et al., 2024	The Influence of Marriage and Cohabitation on Physical Activity Among Middle-Aged and Older People.	Age criteria not met
Zhou et al., 2024	The influence of tai chi on the death anxiety of elderly people living alone: the chain mediating effect of social support and psychological capital	No directionality assessed

Note: The term "ineligible anaytical focus" refers to studies in which PA was the predictor, SOSU was the outcome, or both were modelled as independent variables.

Table A31 Quality appraisal: Newcastle-Ottawa-Scale (NOS) for cross-sectional studies

**Author, Year**	**Selection**	**Comparability**	**Outcome**	**Evaluation**
Abdi & O'Hern, 2025	3	2	2	*Good*
Bakhtari et al., 2019	4	2	2	*Good*
Blakoe et al., 2023	4	2	2	*Good*
Bopp et al., 2004	4	2	2	*Good*
Carlson et al., 2012	4	2	2	*Good*
Chan et al., 2020	5	2	3	*Good*
Chen et al., 2021	5	2	3	*Good*
Corseuil Giehl et al., 2017	4	2	2	*Good*
Cousins, 1996	2	2	2	*Fair*
De Sousa et al., 2021	4	2	2	*Good*
Gomes et al., 2017	4	2	2	*Good*
Gothe, 2018	4	2	3	*Good*
Hwang & Yi, 2025	3	1	3	*Good*
Jin et al., 2022	5	2	2	*Good*
Kang et al., 2018	4	3	2	*Good*
Kaplan et al., 2001	4	2	2	*Good*
Kim & Kosma, 2012	3	2	2	*Good*
Krause et al., 1993	4	2	2	*Good*
Lee & Fan, 2023	3	2	2	*Good*
Lee et al., 2025	4	2	2	*Good*
Lian et al., 1999	4	2	2	*Good*
Lieber et al., 2024	3	2	1	*Fair*
Loprinzi & Joyner, 2016	4	2	2	*Good*
Marthammuthu et al., 2023	4	2	2	*Good*
Newsom et al., 2018	3	2	2	*Good*
Oka & Shibata, 2012	2	2	3	*Fair*
Orsega-Smith et al., 2007	4	2	2	*Good*

Ory et al., 2016	3	2	2	*Good*
Park et al., 2014	4	2	2	*Good*
Perrino et al., 2011	4	2	2	*Good*
Safavi et al., 2025	5	2	2	*Good*
Salvador et al., 2009	5	2	2	*Good*
Shores et al., 2009	5	2	2	*Good*
Sirotiak et al., 2024	2	2	2	*Fair*
Steijvers et al., 2025	3	2	2	*Good*
Thornton et al., 2017	4	2	3	*Good*
Van Cauwenberg et al., 2014	4	2	2	*Good*
Van Luchene et al., 2021	2	2	2	*Fair*
Wagner et al., 2020	4	2	2	*Good*
Watt et al., 2014	4	2	2	*Good*
Wendt Böhm et al., 2016	5	2	2	*Good*
Wilcox et al., 2003	3	2	2	*Good*
Yamakita et al., 2015	4	2	2	*Good*
Yi et al., 2016	4	2	2	*Good*
Yuan et al., 2025	3	2	2	*Good*
Zambarano Garza et al., 2024	2	2	2	*Fair*
Zambarano Garza et al., 2025	2	2	2	*Fair*
Zhou et al., 2023	3	2	1	*Poor*
Zimmer & McDonough, 2021	4	2	2	*Good*

Threshold for converting the NOS for cross-sectional studies: good quality (3 to 5 stars in selection domain AND 1 or 2 stars in comparability domain AND 2 or 3 stars in outcome domain), fair quality (2 stars in selection domain AND 1 or 2 stars in comparability domain AND 2 or 3 stars in outcome domain), poor quality (0 or 1 star in selection domain OR 0 stars in comparability domain OR 0 or 1 stars in outcome domain)

Table A42 Quality appraisal: Newcastle-Ottawa-Scale (NOS) for longitudinal studies

**Author, Year**	**Selection**	**Comparability**	**Outcome**	**Evaluation**
Harvey & Alexander, 2012	4	2	2	Good
Manz et al., 2018	4	2	2	Good
Smith et al., 2023	4	2	2	Good
Komazawa et al., 2021	3	2	2	Good
Warner et al., 2011	4	2	2	Good

Threshold for converting the NOS for longitudinal studies: good quality (3 to 4 stars in selection domain AND 1 or 2 stars in comparability domain AND 2 or 3 stars in outcome domain), fair quality (2 stars in selection domain AND 1 or 2 stars in comparability domain AND 2 or 3 stars in outcome domain), poor quality (0 or 1 star in selection domain OR 0 stars in comparability domain OR 0 or 1 stars in outcome domain)
