# Supplementary material for: Functional social support and physical activity in community-dwelling older adults: a systematic review and meta-analysis
Source: BMC Public Health. 2026 Sep 1;26:2504. doi: 10.1186/s12889-026-28972-z (PMC13536801; doi:10.1186/s12889-026-28972-z)
Supplement: Supplementary file 2 [file 12889_2026_28972_MOESM1_ESM.docx]

**Supplementary Material 1**

**Search terms**

"aging" OR "ageing" OR "old* adult*" OR "late* life" OR "elder*" OR "geriatric*" OR "old* people" OR "old* male*" OR "old* female*" OR "late-life" OR "old* patient*" OR "old* age" OR "late adulthood" OR "life span" OR "life course" OR "second half of life" OR "life-span" OR "life-course" OR "aged" OR "old* person*" OR "lifespan" OR "old* population*" OR "HRS" OR "MHAS" OR "ELSA" OR "SHARE" OR "CRELES" OR "KLoSA" OR "JSTAR" OR "TILDA" OR "CHARLS" OR "LASI" OR "MARS" OR "IFLS" OR "SAGE" OR "HAALSI" OR "HAGIS" OR "NICOLA" OR "ELSI" OR "HART" OR "Health and Retirement Study" OR "Mexican Health and Aging Study" OR "English Longitudinal Study of Ageing" OR "Survey of Health, Ageing and Retirement in Europe" OR "Costa Rican Longevity and Healthy Aging Study" OR "Korean Longitudinal Study of Aging" OR "Japanese Study of Aging and Retirement" OR "The Irish Longitudinal Study on Ageing" OR "China Health and Retirement Longitudinal Study" OR "Longitudinal Aging Study in India" OR "Malaysia Ageing and Retirement Survey" OR "Indonesia Family Life Survey" OR "Study on Global Ageing and Adult Health" OR" Health and Aging Study in Africa" OR" Healthy Ageing in Scotland" OR "Northern Ireland Cohort for the Longitudinal Study of Ageing" OR "Brazilian Longitudinal Study of Aging" OR "Health, Aging, and Retirement in Thailand"

AND

"social support" OR "social network*" OR "social relation*" OR "social contact*" OR "social isolation" OR "social capital" OR "lonel*" OR "social engagement" OR "social integration" OR "social activit*" OR "social withdrawal" OR "social participation" OR "social disengagement" OR "personal network*" OR "social tie*" OR "social interaction" OR "social embeddedness" OR "family relation*" OR "kinship relation*" OR "friendship*" OR "social influence*" OR "social vulnerability" OR "peer support" OR "emotional support" OR "social connectedness" OR "belongingness" OR "socially isolated" OR "social environment" OR "tangible support" OR "emotional closeness" OR "informational support" OR "instrumental support" OR "appraisal support" OR "companionship support" OR "functional support" OR "structural support" OR "perceived support" OR "received support" OR "practical support" OR "esteem support" OR "confident support"

AND

"physical* activ*" OR "PA" OR "physical exercis*" OR "exercis*" OR "training" OR "fitness" OR "sport*" OR "physical* inactiv*" OR "aerobic exercis*" OR "run*" OR "swim*" OR "walk*" OR "cycl*" OR "bicyl*" OR "biking" OR "bike" OR "MVPA" OR "LTPA" OR "sedentary"
